# Predicting protein function and orientation on a gold nanoparticle surface using a residue-based affinity scale

**DOI:** 10.1038/s41467-022-34749-w

**Published:** 2022-11-27

**Authors:** Joanna Xiuzhu Xu, Md. Siddik Alom, Rahul Yadav, Nicholas C. Fitzkee

**Affiliations:** Department of Chemistry, Mississippi State University, Mississippi State, MS 39762 USA

**Keywords:** Nanoparticles, Molecular conformation, Solution-state NMR

## Abstract

The orientation adopted by proteins on nanoparticle surfaces determines the nanoparticle’s bioactivity and its interactions with living systems. Here, we present a residue-based affinity scale for predicting protein orientation on citrate-gold nanoparticles (AuNPs). Competitive binding between protein variants accounts for thermodynamic and kinetic aspects of adsorption in this scale. For hydrophobic residues, the steric considerations dominate, whereas electrostatic interactions are critical for hydrophilic residues. The scale rationalizes the well-defined binding orientation of the small GB3 protein, and it subsequently predicts the orientation and active site accessibility of two enzymes on AuNPs. Additionally, our approach accounts for the AuNP-bound activity of five out of six additional enzymes from the literature. The model developed here enables high-throughput predictions of protein behavior on nanoparticles, and it enhances our understanding of protein orientation in the biomolecular corona, which should greatly enhance the performance and safety of nanomedicines used in vivo.

## Abstract (web summary)

The orientation of proteins on nanoparticle surfaces is important to the nanoparticle’s fate in vivo. Here, the authors use competitive binding between protein variants to develop a residue-based affinity scale to develop a model for the binding and orientation of proteins on gold nanoparticles

## Introduction

Understanding the behavior of surface-adsorbed proteins on nanoparticles (NP) is crucial when NPs are used in vivo as a therapeutic drug carrier, phototherapeutic agent, or imaging tool^1–4^. There is increasing evidence that the composition of the adsorbed proteins is not as important as their structure and orientation^5–7^, as the latter governs the three-dimensional presentation of proteins for molecular recognition, which will define the biological performance and fate of the NP^8–10^. For example, the arrangement and orientation of the adsorbed proteins on a NP define the distribution of epitopes presented for immune response^5^ and determine the success of cell targeting^6,9^. Indeed, steric hindrance from the NP surface can restrict access to the important sites of the adsorbed protein, preventing its molecular recognition^11^. In this regard, various methods of controlling the bound protein orientation have been developed for retaining protein bioactivity and enhancing targeting efficiency of protein-NP conjugates^10,12–14^. While knowing the bound orientation is critical to predicting the fate of the NP, obtaining this information experimentally is a daunting task. Only a handful of NMR techniques can probe the NP-binding sites of a protein in situ^15,16^, but these methods have substantial technical challenges and limitations^17^.

In this work, we hypothesize that protein-NP association is mediated primarily by the surface residues of a protein. Therefore, we generate an experimental affinity scale for each residue by varying a key binding position in the small GB3 protein^18^. Competitive protein binding is required to rationalize residue differences^19,20^, demonstrating that both kinetic and thermodynamic considerations influence adsorption^21–23^. This affinity scale and a simple surface-area based algorithm predict the preferred orientation/function for three proteins presented here, including two blind predictions; moreover, our findings rationalize the observed function of five additional proteins from the literature. The presented approach is widely applicable to different nanoparticle systems to improve performance and biosafety, and it enables high-throughput predictions in large protein datasets.

## Results

### A residue-based affinity scale for AuNP binding

**Fig. 1:**
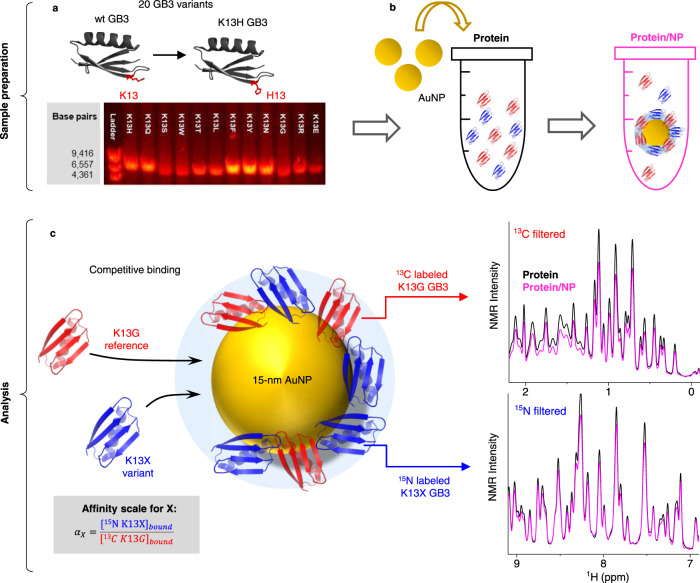
Determination of affinity scales for 20 amino acid residues with host-guest competitive binding experiments. **a** Mutagenesis of K13 into each of the 20 amino acids with histidine (H13) as an example. The agarose gel electrophoresis shows several examples of PCR products for GB3 mutants. **b** Preparation of competitive binding experiments. Two GB3 variants (protein) are mixed before adding AuNPs (protein/NP sample), and a protein corona is formed upon adsorption. **c** NMR analysis for quantification of binding capacity of each variant. The reference variant ^13^C labeled K13G (red) and ^15^N labeled K13X (blue) is measured with ^13^C filtered and ^15^N filtered 1D NMR, respectively. The bound concentration of each variant is deduced from NMR signal reduction from the protein sample (black) and protein/NP sample (magenta) prepared in **b**. Finally, the affinity scale (*α*_*X*_) for residue X is the ratio between the bound concentrations of K13X ([^15^*N* K13*X*]_*bound*_) and K13G ([^13^*C K*13*G*]_*bound*_).

We began by systematically examining a series of GB3 variants where the 13^th^ position was changed to all 20 amino acids (Fig. 1a). Prior work established that the interaction of GB3 with 15-nm citrate-capped AuNP is mediated by lysine residues^18,24^. In particular, K13 contributes most to the binding, and mutation of this residue to alanine significantly reduces GB3 binding^18^. None of the variants had significantly altered structure or stability as assessed by NMR (Supplementary Information). To measure the binding affinity of residue X in regard to the simplest residue glycine (G), an equal amount of K13X GB3 was mixed with K13G GB3 before exposing to AuNPs for competitive binding (Fig. 1b, c). After equilibrium is attained (1 h), the reduction in NMR signals corresponds to the amount of GB3 in the hard corona, as all signals from the bound proteins are broadened beyond detection (Fig. 1c, right)^19^. To disentangle NMR signals of the protein mixture, variant K13X GB3 is ^15^N-labeled, whereas the reference variant K13G GB3 is ^13^C-labeled. This enables in situ quantification of the bound amount of each variant, and the affinity scale for all 20 amino acid residues. The *α* value is defined as the ratio of the K13X variant bound relative to K13G (Table 1). Thus, *α* is derived using non-disruptive, in situ measurements, where the corona is formed in the presence of protein competition.

**Table 1:** Alpha values of 20 GB3 variants

Category	Variant	α	Uncertainty* (95%CI)
Small	K13G	0.98	0.04
Hydrophobic	K13A	0.66	0.04
	K13L	0.40	0.04
	K13I	0.40	0.04
	K13V	0.46	0.04
	K13M	0.74	0.04
	K13P	0.63	0.04
Aromatic	K13F	0.46	0.04
	K13Y	0.47	0.04
	K13W	0.41	0.04
Nucleophilic	K13S	0.57	0.04
	K13C	5.69	0.04
	K13T	0.50	0.04
Acidic	K13E	0.35	0.04
	K13D	0.47	0.04
Amide	K13N	0.54	0.04
	K13Q	0.40	0.04
Basic	K13H	0.79	0.04
	K13K (wt)	0.81	0.04
	K13R	0.84	0.04

*Note: The uncertainties represent 95% confidence intervals as described in the Supplementary Information.

The fact that *α*_*G*_ (^15^N K13G vs. ^13^C K13G) equals 1 (*α*_*G*_ = 0.98 ± 0.04) indicates different isotopic labeling has no measurable impact on the GB3-AuNP interaction. Cysteine has the highest affinity towards AuNP (*α*_*C*_ = 5.69 ± 0.04), due to strong Au-S bond formation^25^. All other α values are <1, regardless of electrostatic, geometric, or hydrophobic properties of a residue. This is surprising, and it suggests that reducing steric bulk and maximizing protein-surface contact can be advantageous to adsorption. The fact that the smallest side chain renders K13G GB3 highest affinity in competition led us to evaluate the role of the steric effect afforded by different residues.

**Fig. 2:**
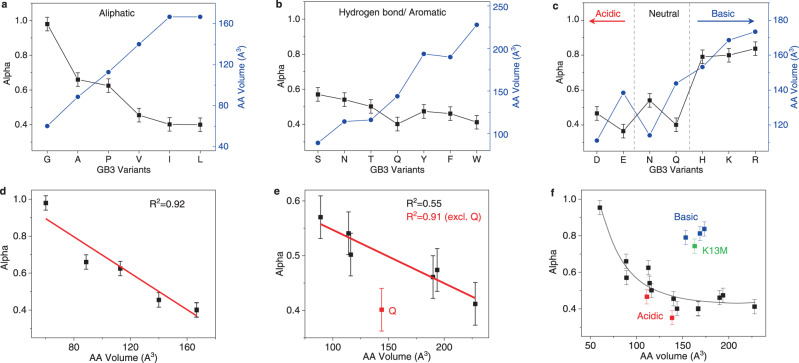
Effect of residue identity on binding affinity. Correlation of *α* value (black) and amino acid (AA) molecular volumes^69^ (blue) for K13X variants with aliphatic side chains (**a**), hydrogen-binding/aromatic side chains (**b**), and electrostatic side chains (**c**). The red arrow and blue arrow indicate increasing acidity and basicity. **d**, **e** Linear fitting (red line)of alpha values as a function of AA volumes for variants presented in **a** and **b**. **f**, Relationship of alpha values and AA volume for all 20 GB3 variants. The gray curve is a polynomial fit to guide the eye. Data points of acidic, basic residues and K13M are highlighted in red, blue and green, respectively. R^2^ is calculated from Pearson’s correlation coefficient using OriginPro Software. All error bars are an averaged 95% confidence interval error as described in the Supplementary Information (*n* = 3 independent experiments).

Indeed, the *α* values correlate inversely with amino acid molecular volumes (AA volume) among K13X GB3 variants modified with aliphatic side chains (Fig. 2a) and hydrogen bonding (H bond)/aromatic side chains (Fig. 2b), with strong linearity observed for each residue sub-group (Fig. 2d, e), except K13Q. This steric effect appears most significant for smaller amino acid side chains. The slopes from the aliphatic subgroup and the polar/aromatic subgroup differ by a factor of five ((−49 ± 7) × 10^−4^ vs. (−9.6 ± 2) × 10^−4^, Fig. 2d, e). Besides this, the hydrophilicity afforded by the terminal hydroxyl group (S, T, Y), or the polarity (W) may play a role in counteracting the increasing steric effect in the polar/aromatic subgroup. Electrostatic interactions can also counter the steric effect, as demonstrated in the charged subgroup (Fig. 2c). While H, K, and R have a ~30% larger volume than D or E, their alpha value is much larger, reflecting a favorable interaction between these residues and the negatively charged citrate-coated surface. This effect is clearly seen in histidine, where increasing pH leads to a steady decrease in *α*_*H*_ (Supplementary Fig. 6). However, even for D and E, an increase in volume correlates with a decrease in alpha (Fig. 2c), and this trend applies for residues with similar functional groups (e.g. Q vs. N). An unexpectedly high *α* is observed for K13M, which may be due to strong attraction between organic sulfides and AuNPs^26^. Overall, increasing side chain volumes appears to reduce competitive binding in our host-guest system, but this effect diminishes for larger side chains (Fig. 2f). Larger side chains on a protein’s surface can sample more rotomeric states, decreasing the protein’s ability to maximize contact with a NP surface, and this may explain the trend we observe.

### Competition is essential for predicting binding affinity

**Fig. 3:**
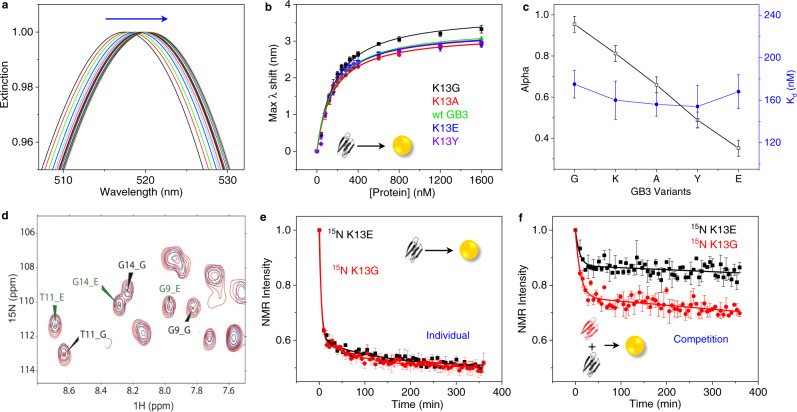
Role of competition in protein corona formation. **a** UV-vis titrations of the K13Y GB3 variant into AuNPs (different colors for each titration point). The arrow represents increasing red shifts of the AuNP peak. **b** Maximum wavelength of the AuNP peak in **a** plotted against protein concentrations for five GB3 variants (K13G, black; K13A, red; WT, green; K13E, blue; K13Y, violet). Each dataset is fit with Langmuir adsorption isotherm model. **c** A comparison of alpha values (black) obtained from competitive binding versus Langmuir dissociation constants (*K*_*d*_, blue). **d** Example SOFAST-HMQC spectrum measuring the competitive binding of K13G (black labels, “_G”) and K13E (green labels, “_E”) to AuNPs. The red spectrum shows a control without AuNPs (0 min), and the blue spectrum is measured with AuNPs at 365 min. Complete spectra are presented in Supplementary Fig. 7. **e** Protein peak intensity change as a function of AuNP incubation time for K13E (black) and K13G (red) GB3. After adding AuNPs, a spectrum was collected every 5 min up to 365 min^19^. An example SOFAST-HMQC NMR spectrum for individual K13E GB3 binding is shown in Supplementary Fig. 8, and individual binding kinetic profiles of other selected variants are shown in Supplementary Fig. 9. A semi-log plot (Supplementary Fig. 10) shows evidence for two decay processes: a fast (<10 min) and a slow (~100–200 min). that a fast and slow decay process. All fitting parameters are shown in Supplementary Table 5. **f** Competitive binding kinetics of K13G versus K13E GB3, with example SOFAST-HMQC spectra presented in **d**. An equal amount of each variant is mixed before addition of AuNPs, rendering total protein concentration double that of the individual binding experiments in **e**. In panels **b** and **c**, error bars are given as the standard error of the mean (SEM) for *n* = 3 i*n*dependent experiments. In panels **e** and **f**, the data are representative, and two independent kinetics experiments were performed to ensure reproducibility. Error bars represent the SEM averaged over *n* ≈ 50 and *n* ≈ 10 resolved peaks, respectively.

Next, we investigated the thermodynamic and kinetic aspects of adsorption as they relate to residue alpha values. First, a single GB3 variant was titrated into an AuNP solution to quantify thermodynamic dissociation constants (*K*_*d*_) of five selected GB3 variants, which represent distinct properties and a range of alpha values: K13G—smallest; K13A—aliphatic; K13K (WT)—basic; K13E—acidic; and K13Y—aromatic. The increasing red shift of the AuNP plasmonic peak is caused by increasing protein adsorption. A common approach is to fit a Langmuir isotherm to these red shifts to extract an apparent dissociation constant, *K*_*d*_ (Fig. 3a, b)^23,27,28^. This approach may be problematic, especially if desorption is slow compared to the measurement timescale; nevertheless, the observed *K*_*d*_ values often mirror other trends in binding affinity^27^. Here, however, there is no statistical difference between thermodynamic *K*_*d*_ values for the GB3 variants. This is in stark contrast to their different α values (Fig. 3c), and the similarity in *K*_*d*_ values likely reflects the fact that adsorption rates are much faster than desorption rates, so that a true equilibrium is not attained. Subsequently, we investigated adsorption kinetics of these variants using time-resolved SOFAST-HMQC 2D NMR spectroscopy, where signal reduction corresponds to protein binding. Here again, no difference was observed for binding kinetics or capacity among these variants when only a single variant was present, e.g. K13G and K13E (Fig. 3e and Supplementary Figs. 9-10). However, when competition is introduced by exposing a mixture of two variants to a solution of AuNPs, K13G was adsorbed faster than K13E and bound to a larger degree, corresponding to its lower alpha value. Even though the initial adsorption rates are too fast to be precisely compared for K13G and K13E (Supplementary Table 5), the alpha value is nevertheless able to differentiate between these variants. Note that only a few well-resolved peaks (e.g., G9, T11, Fig. 3d and Supplementary Fig. 7) can be used for quantification of binding in this mixture (Fig. 3f) due to significant signal overlap^19^, and this approach is somewhat less precise than the ^13^C, ^15^N differential labeling method used above. In these competition experiments, the total protein bound remains the same as when individual variants are bound, supporting the alpha value approach (Fig. 3e vs. f). Together, these results emphasize the importance of competition. In vivo, proteins simultaneously compete for a NP surface, and multiple thermodynamic and kinetic aspects are involved^21–23,29^; the alpha value captures these complexities in one simple metric. Our results also address the limitations of an individual protein binding approach, which may not accurately reflect the corona in complex protein mixtures^7,30,31^.

### Predicting protein bound orientation and function on AuNPs

**Fig. 4:**
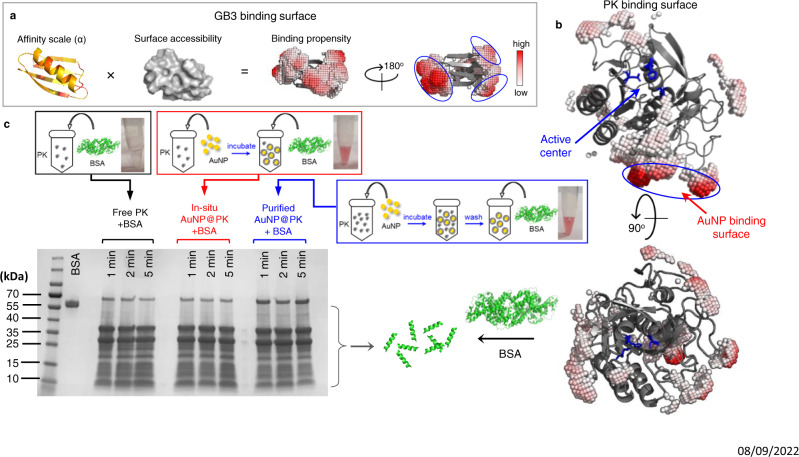
Prediction of bound orientation of Proteinase K (PK) on AuNP. **a** A scheme for deducing the preferred bound surface of GB3 by taking both the alpha value and surface accessibility into account (see main text). Alpha values in the leftmost image are depicted using a yellow (low) to red (high) gradient. The α-helix side and β-sheet side of GB3 show the predicted AuNP-binding surface using color-coded virtual atoms, colored white (low affinity) to red (high affinity). No virtual atoms are drawn below a fixed threshold value. **b** Cartoon structure of PK showing the predicted AuNP-binding surface circled in blue, and its active center constructed by S224, H69 and D39 as highlighted in blue sticks. **c** Preparation and results of PK activity assay for free (black) and AuNP-bound PK. The PK concentration in all assays is kept at 0.01 mg/mL and substrate BSA concentration is at 2 mg/mL for all samples to achieve limited proteolysis. As shown in the scheme (top), before mixing with BSA, the in-situ bound PK sample (in-situ AuNP@PK, red) is prepared by adding AuNPs to bind ~90% of free PK without further purification, whereas the purified AuNP@PK (blue) is washed after incubating excess amount of PK with AuNPs (Supplementary Information). SDS-PAGE shows limited proteolysis of BSA using free PK (lane 4–6), in-situ AuNP@PK (lane 8–10), and pre-coated and washed AuNP@PK (lane 12–14) for reaction times of 1 min, 2 min, and 5 min (from left to right), respectively. Lane 2 shows a 0.2 mg/mL BSA control with no PK. The associated schemes outline their sample preparations, and the photographs show the assay solutions.

Finally, we attempted to predict the preferred AuNP-binding surface of several proteins using the alpha values. We devise a simple metric for the binding affinity of a residue, calculated by multiplying the alpha value with the accessible surface area relative to a fully exposed sidechain^32^ (Fig. 4a). To visualize regions of high binding affinity, a protein is overlaid on a grid, and the average binding affinity from nearby residues is calculated at each surface-accessible grid point. Grid points are represented by virtual atoms, colored by low (white) to high (red) binding affinity, and values below a threshold are not shown (full details of this alpha-value approach are provided in Supplementary Information, and scripts are available to download on GitHub)^33^. The result for GB3 (Fig. 4a, right) reveals three high-affinity regions on either side of the β-sheet; conversely, the alpha helix has relatively low affinity. Based on this, we predict that GB3 interacts with AuNPs by its β-sheet face, and this orientation is consistent with previous work^18^.

Subsequently, we tested our approach for predicting the orientation of unknown proteins. We chose two enzymes for this purpose, and we hypothesized that enzyme activity could be used to monitor protein orientation indirectly: assuming that a protein remains folded and packed on the NP surface^24^, a bound orientation with a sterically hindered active site should exhibit reduced activity^6,13^. Conversely, an orientation that exposes the active site should have near-normal activity. Proteinase K (PK) has a catalytic triad constructed by S224, H69 and D39, and it non-specifically cleaves peptide bonds in a protein (active site highlighted in blue, Fig. 4b). Our model predicts that PK should bind to AuNPs through the residues highlighted by the red binding patches (Fig. 4b), leaving its substrate access sterically unaffected. We examine this prediction by quantifying the proteolytic efficiency of NP-bound PK for digesting bovine serum albumin (BSA). With a size of 66 kDa, BSA should be sensitive to any steric hindrance toward the substrate-binding site of PK. For rigorous validation, the AuNP-bound PK (AuNP@PK) was prepared in two ways: in-situ, where AuNPs are simply mixed with PK without further perturbation; and washing, where AuNP@PK was centrifuged and resuspended three times in buffer to ensure removal of free PK from the solution (purified AuNP@PK). The purified, precoated AuNP@PK was characterized by UV-vis spectrometry and DLS (Supplementary Fig. 11) to ensure no AuNP aggregation. The activities of both in-situ and purified AuNP@PK were compared with a control sample of PK with no AuNPs, and the total PK concentration was held constant (Supplementary Information). In both cases, the nanoparticles are fully saturated with PK before mixing with BSA; otherwise, BSA may bind and be protected from proteolysis^34,35^. This protection was observed when AuNPs in large excess (~4 times more AuNP surface) were added to conjugate a limited amount of PK, followed by mixing with BSA (Supplementary Fig. 12).

After optimizing the enzyme/substrate ratio for limited proteolysis of BSA within a 1–5 min observation time, we found that PK exhibits identical proteolytic efficiency in both free and AuNP-bound (AuNP@PK) forms (Fig. 4c). In particular, the band intensities corresponding to the cleavage of intact BSA into smaller peptides are almost identical. Adding AuNPs to PK in the in-situ experiment shows no reduced activity, and washing the AuNP@PK particles before mixing with BSA confirms that the activity originates from the on-AuNP PK. This suggests that the orientation of PK on the AuNP surface matches our prediction, with no steric hindrance to its active site (Fig. 4b).

**Fig. 5:**
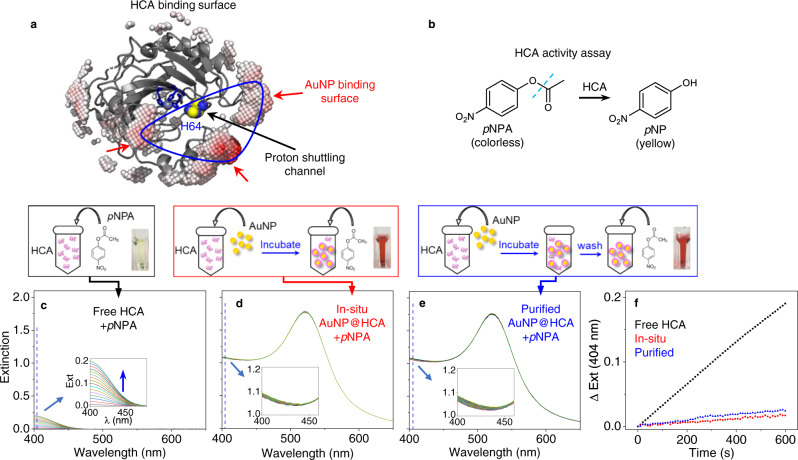
Prediction of bound orientation of Human carbonic anhydrase (HCA) on AuNP. **a** Cartoon structure of HCA showing the predicted AuNP-binding surface (highlighted blue region) with virtual atoms, with respect to its active center (highlighted in blue sticks), proton shuttling channel, Zn^2+^(gray sphere) and H64 (highlighted blue and yellow spheres). **b** Mechanism of HCA assay of hydrolyzing colorless *p*NPA to yellow *p*NP, which absorbs at 404 nm. **c**–**e** Time-resolved UV-vis spectra of HCA assay solutions with incubation time of 10 min using free HCA (black, **c**), in-situ HCA bound to AuNPs (AuNP@HCA, red, **d**), and washed/purified AuNP@HCA (blue, **e**). The enzyme/substrate ratio is fixed at 0.2 µM HCA/100 µM *p*NPA in all assays. The associated schemes above illustrate the sample preparation, and pictures show each solution after 30-min incubation. **f** Comparisons of extinction change at 404 nm as a function of reaction time using free HCA (black), in-situ AuNP@HCA (red) and purified AuNP@HCA (blue). Close-up UV-Vis spectra are shown as insets in **c**–**e**.

Using this same approach, we predict that the AuNP binding surface and substrate-binding site will be coincident in human carbonic anhydrase II (HCA). As depicted in Fig. 5a, the HCA active center is constructed by Zn^2+^ (gray sphere), H96, H94, and H119 (highlighted in sticks)^36^. During the conversion of carbon dioxide into carbonic acid by HCA, residue H64 (showed as sphere) plays an essential role in shuttling protons from the active center to solvent^37^. The binding of HCA onto AuNP is predicted to occlude the active center and prevent proton transportation by H64 to the environment. We tested the activity of HCA in the free and bound states using a *p*-nitrophenyl acetate (*p*NPA) hydrolysis assay (Fig. 5b), where NP-bound HCA (AuNP@HCA) was prepared in-situ and by AuNP washing, similarly to PK. Free HCA converts *p*NPA to *p*NP rapidly and efficiently upon mixing, as indicated by the increasing absorbance from *p*NP at 404 nm (Fig. 5c). However, on AuNPs, the activity of the bound HCA is mostly inhibited regardless of preparation method (Fig. 5d, e). Prior work by Saada et al. suggests that this inactivation occurs mainly due reduced accessibility of H64 rather than denaturation of HCA by AuNP^38^. In their work, the authors show that introducing functional groups with histidine-like imidazole rings on AuNP could support the activity of the adsorbed HCA. This result also suggests the channel of proton shuttling is oriented toward the AuNP surface, as predicted by our model.

The alpha-value approach was developed for 15 nm AuNPs, but it is possible that nanoparticle curvature modulates protein interactions. Curvature alters protein-protein interactions on the surface and can influence protein orientation and stability^39^. To test the effects of curvature, we performed the enzyme activity studies described above on both 10 nm and 30 nm spherical AuNPs (Supplementary Figs. 14-15). For the in situ reactions, where the stoichiometry was controlled so that nearly all of the enzyme was bound to the AuNP, both PK and HCA behaved identically for 10 and 30 nm AuNPs; PK was fully active, and HCA was inactive. These reactions had essentially no excess enzyme in solution, suggesting that our model holds over a moderate range of curvatures. For the purified reactions, where enzyme-coated AuNPs were washed and resuspended using centrifugation, HCA was also inactive. However, PK exhibited slightly reduced activity on 30 nm AuNPs, and more undigested BSA was detected (Supplementary Fig. 14). This could be a result of nanoparticle agglomeration during purification/centrifugation, which occurs more readily for larger AuNPs^40^. The in situ result, which has minimal perturbation, shows complete activity for PK, and it is unlikely that, if the protein is significantly destabilized on the AuNP surface, the small amount of residually active protein will have precisely the same activity as free PK. PK retains significant activity on 30 nm AuNPs, even after washing, and we believe this supports the generality of our model.

Our approach is able to rationalize the enzyme behavior from the literature as well. No steric hindrance of the active site is predicted for two near-fully active AuNP-bound enzymes (acetylcholinesterase^41^ and citrate synthase^41^; all details, along with predicted orientations, are provided in the Supplementary Information). In addition, the predicted orientation of cytochrome c on citrate-reduced silver nanoparticles is consistent with observations by surface-enhanced Raman scattering^42^. In contrast, the activities of bound alcohol dehydrogenase^29^, horseradish peroxidase^43^ and glucose oxidase^44^ are significantly reduced on AuNPs. Our model successfully ascribes the behavior of alcohol dehydrogenase and horseradish peroxidase to partial blockage of the active site; however, the behavior of glucose oxidase is not predicted by our model, which is ascribed to protein unfolding upon adsorption by Lescovac et al.^44^. This highlights one of the limitations of the alpha value approach, namely, that it does not account for decreases in protein stability on a surface^45^.

Overall, this approach will likely work best for stable, globular proteins at concentrations sufficiently high to completely cover the AuNP surface. In other situations, it will perform less well. For example, low protein binding density can cause proteins to deform and induce nanoparticle aggregation^46^, whereas dense adsorption of many proteins is hypothesized to have a stabilizing effect^47^. These observations explain why the alpha value approach works for the cases examined, but its predictive value will be likely limited in highly dilute protein solutions. As another example, unstable and disordered proteins do not form a simple monolayer on AuNPs^45^, and this will also limit the predictive value of alpha for those systems. Furthermore, elongated proteins like fibrinogen can adopt multiple conformations and will not be well predicted by the alpha-value approach. Perry and coworkers have observed multi-step binding for fibrinogen. In their experiments with flat surfaces, side-on binding of fibrinogen occurs first, followed by a rearrangement to end-on binding^48^. While the alpha-value approach predicts that fibrinogen binding will be side-on along the long axis (Supplementary Fig. 16), it cannot account for rearrangements that occur after adsorption. Finally, this approach has been calibrated to citrate-AuNP surfaces and will likely not be applicable to other nanoparticle surface chemistries. For example, polystyrene nanoparticles and silica nanoparticles exhibit a more dynamic corona and can induce significant structural changes to proteins upon adsorption^49,50^. A host-guest approach, similar to what is used here, could also be informative for these types of nanoparticles. However, such an approach would need to be modified to account for reversible binding and protein deformation seen in these systems. Despite these limitations, we believe the alpha-value predictions will be valuable for real-world applications in plasmonic AuNPs; for example, human blood plasma is highly concentrated (60–80 mg/mL), and many of the most abundant proteins by mass are globular^51^.

Computational methods have been used to derive amino acid binding scales to gold^52–57^. Not surprisingly, these do not correlate well with the alpha values presented here (Supplementary Fig. 17). Indeed, many of these scales do not correlate well with each other. There are several possible explanations: First, most potentials of mean force have been derived from data on individual amino acids or small peptides, which are substantially smaller and more flexible than GB3. Secondly, most simulations observe dynamic interactions with amino acids, where adsorption and desorption occur in a single simulation. We and others observe a static hard corona for AuNPs^24,58,59^, introducing the element of kinetic control in adsorption. Finally, many computational scales are calibrated to a bare gold surface, whereas our AuNPs are coated with citrate and have a negative zeta potential. This electrostatic component is significant in protein adsorption^18^, but it will not be reflected for residue scales using a neutral gold surface. A 2015 molecular dynamics (MD) simulation by Menziani and coworkers predicts the orientation of insulin on citrate-coated AuNPs^60^. If the high contribution of Cys residues is excluded from the alpha value (because all Cys residues in insulin form disulfide bonds), there is good agreement between the alpha-value predicted surface and the binding surface from this MD simulation (Supplementary Fig. 18). Understanding the molecular basis of protein-AuNP interactions remains an extremely challenging problem, and the experimentally derived alpha value predictions provide an additional dataset for calibrating simulations.

## Discussion

We have determined an AuNP-binding affinity scale for each amino acid residue using a host-guest approach. Competition is an essential feature of this approach, because protein adsorption to citrate-AuNPs exhibits elements of both thermodynamic and kinetic control. We have demonstrated that this affinity scale can be used to map the residue-specific contribution to AuNP-binding for a protein, and therefore predict its bound orientation and function. This prediction is confirmed experimentally with two representative enzymes. This algorithm can also rationalize the known orientation of the GB3 protein on AuNPs, as well as five out of six other examples from the scientific literature. These results are significant given the biological relevance of a protein’s orientation when bound to NPs. Until now, a straightforward algorithm to predict bound protein activity has not been available; such an algorithm should enable high-throughput predictions. Moving forward, this algorithm can potentially predict the composition of the protein corona formed in vivo by calculating the rank-order binding affinity of various blood proteins in serum. Such a prediction could have tremendous value in the development of nanomedicines with improved safety and functionality.

## Methods

### Chemicals and materials

Gold (III) chloride trihydrate (product #: 520918) and sodium citrate dihydrate (product #: 567446) were purchased from Millipore Sigma. The NMR reference compound deuterated 3-1-propanesulfonic acid-d6 sodium salt (DSS-d6, DLM-8206), Tryptophan (^15^N labeled, NLM-800-PK), and 99.9% D_2_O (DLM-4) were purchased from Cambridge Isotope Labs (Tewksbury, MA). All chemicals were used as purchased without additional purification. Proteinase K (PK) was obtained from Amresco (#0706, biotechnology grade). Bovine serum albumin (BSA) was purchased from Calbiochem (# 12657, electrophoresis 100%). Human carbonic anhydrase II (HCA) was provided by Dr. Joseph P. Emerson at Mississippi State University. Before use, HCA was converted to apo-HCA by dialysis with ACES buffer containing dipicolinic acid at pH 7 described previously^61^. *p*-Nitrophenyl acetate (*p*NPA) was purchased from Sigma-Aldrich (#N8130). All compounds purchased commercially were used without additional purification.

### Synthesis and characterization of 15-nm AuNPs

The method developed by Frens and Turkevich was employed to synthesize AuNPs^62,63^. All glassware was cleaned with aqua regia and thoroughly rinsed with ultrapure water prior to each use. Briefly, 2 mL of 10 mg mL^−1^ HAuCl_4_ was added into 198 mL of 18.2 MΩ ultrapure Milli-Q water in a round bottom flask equipped with a condenser. The reaction was constantly stirred at 750 rpm and heated to boil at power level 70 in a heating mantle (Glas-Col). Once the boiling started, the power level of the mantle was set to zero. When the boiling stopped with no bubbling, 6.5 ml of 1% (w/v) sodium citrate was added, and the power level of the heating mantle was set to 48. The solution was heated at this new power level for 25 min. Subsequently, the flask was removed from the heating mantle and allowed to cool down with constant stirring. When the reaction reached room temperature, 800 µL of 0.5 M sodium citrate was added to stabilize the newly synthesized AuNPs. The synthesized AuNPs were characterized by DLS and UV-vis spectrometry (Supplementary Fig. 1) to ensure their sizes are 15 nm with polydispersity within 10%. Prior to preparation of NMR samples, the AuNP solution was centrifuged in 50 mL Falcon tubes at 8,000 *g* for 45 min at 15 °C. Afterward, the supernatant was removed carefully to concentrate the AuNPs from ~2 nM to 200–300 nM. The exact AuNP concentration was measured by diluting 1 µL of the concentrated AuNP solution into a 1000 µL solution, and determined by its UV-vis peak absorption at 520 nm using a molar absorptivity of 3.94 × 10^8^ M^−1^cm^−1^ ^62^.

### Expression and purification of GB3 variants

The mutant DNA plasmids extracted from XL1 Blue cells were transformed into BL21(Star) DE3 for protein expression (see Supplementary Methods). Briefly, the mutant Bl21(Star) DE3 cells (Invitrogen) and grown for 6 h in 5 mL ampicillin containing LB medium were inoculated into 25 mL ampicillin-containing ^15^N M9 media (starter culture) and grown overnight at 37 °C. The starter culture was then transferred into a 1 L ^15^N M9 media to reach an initial OD_600_ of 0.05. The culture flasks were incubated in a shaker at 37 °C and at 200 rpm and induced with 0.5 ml of 1 mM isopropyl β, D-1-thiogalactopyranoside (IPTG) when the OD_600_ reached 0.5–0.7. After induction, cells were allowed to express protein for ~5 h at 37 °C temperature, and OD_600_ was monitored every hour. Cells were harvested by centrifugation at 7000 *g* for 20 min and cell pellets were collected for the next step (or stored at −80 °C if not used immediately). The harvested cells were resuspended in 20 mL cold lysis buffer (50 mM NaCl, 20 mM NaH_2_PO_4_ pH 4.5, 5 mM EDTA, 0.5 mg/mL lysozyme) on ice. The resuspended cells were sonicated on ice at 45% power for 6 min of total processing time (30 s pulse, 30 s rest). After lysis, the lysed cell solution was incubated in an 85 °C hot water bath for 15 min to allow other cellular proteins to unfold and precipitate, with swirling every 3–4 min. Afterward, the solution was immediately transferred to an ice water bath, and streptomycin sulfate was added (0.5 mg/mL final concentration) to precipitate DNA. After 15 min on ice, denatured proteins and DNA were removed by centrifugation at 18,000 *g* for 45 min. The supernatant was collected and loaded on an AKTA purification system using a 5 mL HiTrap Q FF anion exchange column for purification. Under these conditions (pH 4.5), the column binds additional DNA impurities, while GB3 is flows through in the wash. Fractions with high UV-vis absorption at 280 nm were collected and pooled. The collected fractions were concentrated using 3.0 kDa size EMD Millipore Amicon Ultra Centrifugal Filters (Pall Corporation). For K13C GB3, a final concentration of 5 mM DTT was added to all buffers.

The concentrated protein was further loaded to a HiLoad 26/600 Superdex 75 pg gel filtration column equilibrated with gel filtration buffer (50 mM NaCl, 20 mM NaH_2_PO_4_ pH 4.5). The fractions of K13X GB3 were found to elute around 210 mL with a stronger absorbance at 280 nm than 260 nm. The purified K13X GB3 protein was dialyzed in 10 mM HEPES buffer at pH 6.5 and 5 mM NaCl for 5 h (plus 1 mM TCEP for K13C). All GB3 variants were analyzed by gel electrophoresis to ensure purity (estimated to be >98%).

### Characterization of GB3 variants by 2D TOCSY-HSQC NMR

HSQC, NOESY-HSQC, and TOCSY-HSQC experiments^64^ were performed to assign and backbone amide peaks. A 500 μM ^15^N K13X GB3 protein sample was prepared in HEPES at pH 6.5 and 6% D_2_O for the measurement (with 1 mM TCEP for K13C GB3). The vast majority of GB3’s 56 backbone amide peaks exhibit only a marginal chemical shift perturbation when position 13 is changed; this demonstrates that mutagenesis does not significantly change the folded structure of GB3. The ^15^N and ^1^H chemical shifts of each variant are presented in Supplementary Tables S6-S12.

### Preparation of competitive binding samples of two GB3 variants

Stock solutions of ~150 µM GB3 variants, 100 mM HEPES buffer at 6.5, 500 mM NaCl solution, and ~300 nM AuNP solution were prepared. The extinction coefficient for each variant was determined using the approach of Pace, et al.^65^. To set up competitive binding experiments for each variant, a protein/AuNP mixture (P/NP) NMR sample of a total volume of 400 µL was made with 20 µM ^13^C K13G GB3, 20 µM ^15^N K13X GB3, 20 mM HEPES buffer at pH 6.5, 20 mM NaCl, 18.2 MΩ ultrapure Milli-Q water and 50 nM AuNPs. A protein (P) sample as control was made identically except without the addition of AuNPs. All binding experiments were conducted under pH 6.5 unless indicated otherwise. 100 mM NaOAc/AcOH buffer at pH 5.0 and 100 mM HEPES at pH 8.0 are used instead for pH-dependent binding experiments. The protein/AuNP sample was incubated for an hour before NMR experiment to ensure the binding had reached equilibrium. 4 mM 4,4-dimethyl-4-silapentane-1-sulfonic acid (DSS) was used as an external standard for peak intensity calibration.

### Affinity scale for residue X quantified by 1D filtered NMR experiments

Proton 1D NMR spectra were recorded on a 600 MHz Bruker Avance III cryoprobe-equipped NMR spectrometer with a jump-return program for water suppression described previously^18,24^. An acquisition time of 80 ms was used, and the inter-pulse delay of 110 µs was selected for proton resonances at 8.5 ppm. The total experiment time was 9 min. Three 1D spectra were collected for each sample: A non-filtered experiment for calibrating peak intensities of the DSS reference between the Protein/NP and Protein samples; A ^13^C filtered experiment to obtain the signals from ^13^C K13G GB3 only; A ^15^N filtered experiment to measure the peak intensities solely from ^15^N K13X GB3 (Fig. 1). The binding of GB3 onto AuNP has proven to be in slow exchange regime, and therefore the NMR signal of bound proteins will be invisible due to peak broadening and the remaining signal is attributed to the unbound protein. By comparing the protein NMR signal without (P sample) and with (P/NP sample) the presence of AuNP, the bound amount of ^13^C K13G GB3 and ^15^N K13X GB3 can be determined, respectively. The data processing was performed with TOPSPIN. All 1D NMR spectra are phase adjusted identically and baseline subtracted. The DSS peak intensity was used as a reference to normalize all NMR spectra before comparison. The “scale spectrum” in the TOPSPIN was then used to determine the percentage of integrated proton NMR signal has dropped after the addition of AuNPs, which corresponds to the bound percentages of each variant (Fig. 1). Finally, the alpha value (*α*) of K13X GB3 over K13G GB3 was quantified using Eq. 1. An *α*-value > 1 indicates K13X GB3 binds stronger than K13G GB3 when both are present competing for limited AuNP surface, and vice versa.

1 $$\alpha=\frac{{[{\,\!}^{15}NK13X]}_{bound}}{{[{\,\!}^{15}CK13G]}_{bound}}$$

where *α*_*X*_ is the affinity for residue X, [^15^*N K*13*X*]_*bound*_ and [^13^*C K*13*G*]_*bound*_ represent the bound concentrations of ^15^N-K13X GB3 and ^13^C-K13G GB3, respectively. Additional details about the statistical treatment of alpha values are described in the Supplementary Methods.

### Binding kinetics of GB3 onto AuNP monitored by SOFAST-HMQC

After characterization of binding by UV-visible titrations (see Supplementary Methods), the kinetics of individual or competitive binding of GB3 variants were monitored by SOFAST-HMQC 2D NMR (Bruker parameter set: sfhmqcf3gpphiasi)^66,67^. The parameters used include 32 points in the indirect dimension, acquisition time of 15 ms, 16 scans, and a recycle delay of 100 ms. The samples were prepared as follows: 20 µM K13X GB3 is prepared in 20 mM HEPES buffer and 20 mM NaCl as the Protein sample for “0 min point”. The Protein/NP sample for kinetics measurement was prepared identically as the Protein-only sample, but with addition of 50 nM AuNP. Immediately after mixing the protein and nanoparticle solutions, the Protein/NP sample was vortexed and transferred to an NMR tube with the ^15^N-Trp reference insert^19^, and the tube was loaded into the NMR for shimming and measurement. The Protein-only sample was used for 3D shimming, while 1D-shimming was used for the Protein/NP due to its time sensitivity. Including mixing and shimming, the dead time of the experiment is ~5 min before spectral acquisition of the Protein/NP sample. The acquisition of one SOFAST-HMQC spectrum is around 5 min. A series of SOFAST-HMQC spectra were taken every 5 min for a total time of 365 min.

### Surface prediction for AuNP binding using alpha values

Surface predictions were calculated using in-house scripts using the Python programming language. The BioPy library and NACCESS program^32^ were used to estimate accessible surface area. The output is visualized using PyMOL (Schrodinger). This script, and all others, are maintained at the Fitzkee Lab GitHub repository (https://github.com/FitzkeeLab/citrate-aunp-predict)^33^. Step-by-step procedures for running this scripts are described in the Supplementary Methods.

### Proteinase K proteolytic activity assay

Proteinase K (PK) from *Tritirachium album* (Amresco #0706) was dissolved in 10 mM KH_2_PO_4_ at pH 7.5 and applied to a desalting column (Thermo Scientific, #89882) before use. Its concentration in mg mL^−1^ was determined by UV absorption at 280 nm (ε_280_ = 36,580 M^−1^ cm^−1^) and molecular weight (M.W. = 28,907 g/mol). The activity of PK was evaluated by its proteolytic reaction with BSA as the substrate. Different PK/BSA ratios were explored for incomplete BSA digestion, and 0.01 mg/mL PK and 2 mg/mL BSA was finally used for the assay condition. Initial characterization of PK-functionalized AuNPs was performed to determine the number of proteins bound per AuNP (the binding capacity)^24^. Details are provided in the Supplementary Methods, and the binding capacity of PK (*C*_*PK*_) was determined as 82 ± 6 PK per AuNP (Supplementary Fig. 4).

The AuNP-bound PK was prepared in two ways, in situ (in-situ AuNP@PK) and by washing (purified AuNP@PK). For the in situ method, based on its *C*_*PK*_, 6.8 nM AuNP was mixed with 0.01 mg/mL PK for at least 3 h to ensure that ~90% PK proteins are tightly bound before adding BSA. We make sure all AuNP surfaces are saturated (leaving ~10% excess PK unbound) to avoid BSA binding to AuNP, which would effectively protect BSA from proteolysis. For the washing method, an excess amount of 20 µM PK was mixed with 50 nM AuNPs for 3 h to reach binding equilibrium (solution volume of 400 uL), and the PK-coated AuNPs (AuNP@PK) was spun down by centrifugation at 9000 *g* for 15 mins. The supernatant was carefully removed, and the pellet was washed with 1 mL buffer. The washing process was repeated twice, and the concentration of purified AuNP@PK was characterized to be 12.2 nM using ε_520_ = 3.945 × 10^8^ M^−1^ cm^−1^. Based on the binding capacity of PK, 67.7 µL of AuNP@PK was used for the 200 µL proteolytic reaction to ensure total PK concentration is ~0.01 mg/mL, the same value as for the no-NP sample and the in-situ AuNP@PK sample. Activity assays on 10 nm and 30 nm AuNPs were performed in a similar fashion, and details are provided in the Supplementary Methods.

### Human carbonic anhydrase (HCA) activity assay

Two equivalents of ZnCl_2_ were added to apo-HCA for activity measurements. The HCA concentration was determined using *ε*_280_ of 54,000 M^−1^ cm^−1^. A 10 mM *p*NPA (substrate) stock solution was prepared by dissolving *p*NPA in acetonitrile. A 1000 µL activity reaction was run using the following conditions: 0.2 µM HCA (free or bound) was mixed with 100 µM *p*NPA in 10 mM HEPES buffer at pH 7.5 in a disposable cuvette. Once HCA and *p*NPA were mixed, a series of UV-vis spectra (from 400 nm to 650 nm) were taken every 10 s for 10 min. The extinction at 404 nm was monitored as a function of time to evaluate the esterase activity of HCA, as the colorless *p*NPA was converted to yellow 4-nitrophenol (*p*NP *ε*_404_ = 17,300 M^−1^ cm^−1^). Activity assays on 10, 15, and 30 nm AuNPs were performed in a similar fashion, and details are provided in the Supplementary Methods.

### Statistics and reproducibility

For gel electrophoresis data (PCR mutagenesis, proteinase K assays), all gels were collected at least three times to ensure that assays were reproducible. GB3 variants were also sequenced (Eurofins, Louisville, KY) to ensure the correct mutation was present.

### Reporting summary

Further information on research design is available in the Nature Portfolio Reporting Summary linked to this article.

## Supplementary information

Supplementary Information

Reporting Summary

## Data Availability

Protein structures used in this study were obtained from the Protein Data Bank (https://www.rcsb.org/). PDB accession codes are: 2OED (GB3), 5B1E (proteinase K), 1CA2 (carbonic hydrase), 1HCH (horseradish peroxidase), 4ewz (insulin), 1YKF (alcohol dehydrogenase), 1EEA (acetylcholinesterase), 1CTS (citrate synthase), 1OCD (cytochrome *c*), 1GAL (gluocose oxidase), and 3GHG (fibrinogen). All data, including NMR kinetics data, UV-vis spectra, protein activity data, and surface interaction calculations data generated in this study have been deposited in the Zenodo database under accession code 10.5281/zenodo.7272338^68^.

## Code availability

The code used to generate alpha value predictions are available in the GitHub repository, located at https://github.com/FitzkeeLab/citrate-aunp-predict^33^.
