# Supplementary material for: Predicting protein function and orientation on a gold nanoparticle surface using a residue-based affinity scale
Source: Nat Commun. 2022 Nov 27;13:7313. doi: 10.1038/s41467-022-34749-w (PMC9701677; doi:10.1038/s41467-022-34749-w)
Supplement: Supplementary file 1 — Supplementary Information [file 41467_2022_34749_MOESM1_ESM.pdf]

Supplementary Information

Predicting Protein Function and Orientation on a Gold Nanoparticle Surface Using a Residue-Based Affinity Scale

Joanna Xiuzhu Xu [†], *Md. Siddik Alom*[†], *Rahul Yadav*[†] *and Nicholas C. Fitzkee*[†, *]

Table of Contents

Additional Supplementary Methods **2**

Synthesis and characterization of 15-nm AuNPs 2

Site-directed mutagenesis for 20 GB3 variants 3

Characterization of GB3 variants by 2D TOCSY-HSQC NMR. 8

Affinity scale for residue X quantified by 1D filtered NMR experiments 9

UV-vis titration for thermodynamic characterization of GB3 adsorption on AuNP 11

Surface prediction for AuNP binding using alpha values 11

Proteinase K (PK) binding capacity determination 13

Human carbonic anhydrase (HCA) binding capacity determination 14

Proteinase K proteolytic activity assay with 10 nm and 30 nm AuNPs 14

HCA activity assay on 10 nm and 30 nm AuNPs 15

Quantification of Charge Effect on GB3 binding onto AuNPs **17**

Kinetics study of GB3 binding onto AuNPs with SOFAST-HMQC **18**

Characterization of Purified AuNP@PK **22**

Characterization of purified AuNP@HCA **23**

Examination of Enzyme Activities on 10 nm and 30 nm AuNPs **24**

Predicted Interaction Surface of Human Fibrinogen **26**

Comparison with Computational Simulations of Protein-AuNP Binding **27**

Survey of Literature-Reported AuNP-bound Enzyme Activities **29**

^{1}H and ^{15}N Chemical Shifts of 20 GB3 variants **35**

References **49**

Additional Supplementary Methods

Synthesis and characterization of 15-nm AuNPs

Following synthesis (described in the text) 15-nm gold nanoparticles (AuNPs) were characterized by dynamic light scattering (DLS) on an Anton Paar Litesizer 500 as shown below. The lower panel shows a representative UV-vis extinction spectrum.

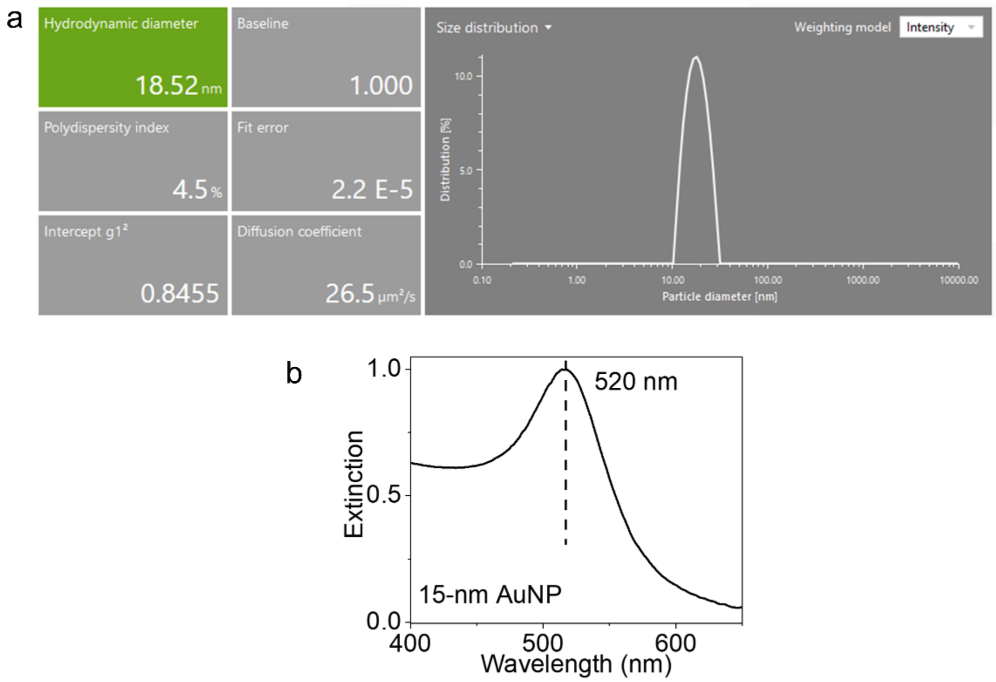

Supplementary Fig. 1 | Characterization of as-synthesized 15-nm citrate-capped AuNPs. a, Particle size distribution determined by Anton Paar DLS system. **b,** UV-vis extinction spectrum of as-synthesized AuNPs.

Site-directed mutagenesis for 20 GB3 variants

Site-directed mutagenesis was performed on the pGS-21 vector of the wild-type (wt) GB3 sequence (**Supplementary Fig. 2**) to introduce K13X point mutation.

```
                                **M**  Q  Y  K  L  V  I  N  G  K  T  L  K     13
1      ctttaagaaggagatatacatatgcagtacaaattagttatcaatggtaaaacattgaaa   60

14     G  E  T  T  T  K  A  V  D  A  E  T  A  E  K  A  F  K  Q  Y     33
61     ggcgaaacaactactaaagctgttgatgctgaaactgcagaaaaagctttcaaacaatac   120

34     A  N  D  N  G  V  D  G  V  W  T  Y  D  D  A  T  K  T  F  T     53
121    gctaacgacaacggtgttgacggtgtttggacttacgacgatgcgactaagacctttaca   180

54     V  T  E  *
181    gttactgaataggatccggctgctaacaaagcc  213
```

Supplementary Fig. 2 | DNA and translated protein sequence of wt GB3. In this figure, the highlighted, bold M represents the start of translation, and the asterisk represents the stop codon. One-letter amino acid codes are shown on the top line in capital letters, and the DNA sequence is shown in the lower line in lower case. Flanking, untranslated nucleotides from the pGS-21 vector are shown in addition to the translated sequence.

Primers were designed and optimized using GeneRunner Software version 6.5.52 (http://www.generunner.net/) and the New England Biolabs (NEB) T_m Calculator (https://tmcalculator.neb.com/#!/main). Parameters including melting temperature, percent of GC content, annealing temperature (T_m), probability of primer dimerization, and the number of nucleotides were optimized. A total of 19 sets of primers used in this study are listed in Supplementary Table 1. The bases in red represent the codons that were changed. However, a single nucleotide in the codon of K10 (AAA to AAG) was also changed for K13F, K13N, K13Y, and K13M mutants. This change does not alter K10, but it increases the GC content and was found to increase the polymerase chain reaction (PCR) success rate.

Site-directed Mutagenesis was performed with PCR using the Phusion High-Fidelity DNA Polymerase kit by NEB (NEB # E0553S). Stock solutions of 10 µM forward (Fwd) and reverse

(Rev) primers were made using 18.2 MΩ ultrapure Milli-Q water. Wild type pGS-21 plasmid was extracted from a 10 ml culture media of *E. Coli* XL1 Blue (Invitrogen) cells using a miniprep plasmid extraction protocol,[3] and a 20 ng/µL stock solution was obtained as the DNA template for PCR. In a typical PCR setup, final concentrations of 0.5 uM Fwd primer, 0.5 uM Rev primer, 200 uM dNTPs, 20 ng template DNA, 1.0 unit of Phusion DNA polymerase were used. Detailed mixing protocols are presented in Supplementary Table 2. Subsequently, the reaction was carried out in a PCR thermocycler (GeneAmp PCR system 9700). The upper thermal blocks of a PCR machine were preheated to 98°C before transferring PCR tubes from ice to prevent condensation on the cap during heating. Thermocycling conditions for the PCR used in this work are given in Supplementary Table 3. The denaturation, annealing, and elongation steps were repeated for 28 cycles.

The PCR product was analyzed on DNA gel. Briefly, 10 uL of PCR product was stained un a gel containing a final concentration of 0.5 ug/mL Ethidium Bromide (EtBr). A 0.5% agarose (Sigma Aldrich) gel was cast, and electrophoresis was performed at a constant 75 V for 90 minutes. The gel was cast using 1 X TAE buffer with 0.5µg/mL EtBr. Once electrophoresis was finished, the gel was carefully transferred to a UV-photographic system for visualizing the bands corresponding to plasmid lengths compared with the standard DNA ladder.

The template DNA was digested by DpnI reaction using the DpnI kit (NEB #R0176L). 1uL of the DpnI restriction enzyme was added to the remaining PCR product (~40 µL after gel analysis), 5 µL NEBuffer, and MQ water to make a 50 µL reaction. Subsequently, the reaction mixture was incubated at 37 ^{o}C in the PCR thermocycler for an hour. A PCR cleanup kit (Wizard SV, Promega) was then used to purify the synthesized DNA after the DpnI reaction.

The purified DNA was transformed to competent *E. Coli* XL1 Blue cells by heat shock DNA transformation.[4] These cells were plated on ampicillin-containing LB media and grown overnight at 37℃. Single colonies from the plates were transferred to ampicillin containing lysogeny broth (LB) media for overnight growth, and plasmids were extracted using the miniprep plasmid extraction protocol (Wizard SV, Promega).[3] To get the final confirmation of a successful mutation, 10 µL purified plasmid was sent for Sanger sequencing (Eurofins Genomics) using the T7 promoter.

Supplementary Table 1 | PCR Primers used in this study

Primer Name	Primer Sequence (5' to 3')
K13R -Fwd	GGTAAAACATTGCGTGGCGAAACAACTACTAAAGCTG
K13R -Rev	GCTTTAGTAGTTGTTTCGCCACGCAATGTTTTACCATTG
K13D -Fwd	GGTAAAACATTGGACGGCGAAACAACTACTAAAGCTG
K13D -Rev	GCTTTAGTAGTTGTTTCGCCGTCCAATGTTTTACCATTG
K13E -Fwd	GGTAAAACATTGGAGGGCGAAACAACTACTAAAGCTG
K13E -Rev	GCTTTAGTAGTTGTTTCGCCCTCCAATGTTTTACCATTG
K13T -Fwd	GGTAAAACATTGACGGGCGAAACAACTACTAAAGCTG
K13T -Rev	GCTTTAGTAGTTGTTTCGCCCGTCAATGTTTTACCATTG
K13V -Fwd	GGTAAAACATTGGTGGGCGAAACAACTACTAAAGCTG
K13V -Rev	GCTTTAGTAGTTGTTTCGCCCACCAATGTTTTACCATTG
K13C -Fwd	GGTAAAACATTGTGCGGCGAAACAACTACTAAAGCTG
K13C -Rev	GCTTTAGTAGTTGTTTCGCCGCACAATGTTTTACCATTG
K13G -Fwd	GGTAAAACATTGGGTGGCGAAACAACTACTAAAGCTG
K13G -Rev	GCTTTAGTAGTTGTTTCGCCACCCAATGTTTTACCATTG
K13I -Fwd	GGTAAAACATTGATCGGCGAAACAACTACTAAAGCTG
K13I -Rev	GCTTTAGTAGTTGTTTCGCCGATCAATGTTTTACCATTG

Primer Name	Primer Sequence (5' to 3')
K13F -Fwd	GGTAAGACATTGTTCGGCGAAACAACTACTAAAGCTG
K13F -Rev	GCTTTAGTAGTTGTTTCGCCGAACAATGTCTTACCATTG
K13Y -Fwd	GGTAAGACATTGTACGGCGAAACAACTACTAAAGCTG
K13Y -Rev	GCTTTAGTAGTTGTTTCGCCGTACAATGTCTTACCATTG
K13N -Fwd	GGTAAGACATTGAACGGCGAAACAACTACTAAAGCTG
K13N - Rev	GCTTTAGTAGTTGTTTCGCCGTTCAATGTCTTACCATTG
K13M -Fwd	GGTAAGACATTGATGGGCGAAACAACTACTAAAGCTG
K13M -Rev	GCTTTAGTAGTTGTTTCGCCCATCAATGTCTTACCATTG
K13A -Fwd	GGTAAAACATTGGCAGGCGAAACAACTACTAAAGCTG
K13A -Rev	GCTTTAGTAGTTGTTTCGCCTGCCAATGTTTTACCATTG
K13L -Fwd	GGTAAAACATTGCTGGGCGAAACAACTACTAAAGCTG
K13L -Rev	GCTTTAGTAGTTGTTTCGCCCAGCAATGTTTTACCATTG
K13S -Fwd	CATTGAGCGGCGAAACAACTAC
K13S -Rev	CAAAGCGGCGCTTTACAAAATG
K13H -Fwd	CATTGCACGGCGAAACAACTAC
K13H -Rev	CAAAGCGGCGTGTTACAAAATG
K13W-Fwd	CATTGTGGGGCGAAACAACTAC
K13W -Rev	CAAAGCGGCCCATTACAAAATG
K13P-Fwd	CATTGCCGGGCGAAACAACTAC
K13P -Rev	CAACAAAGCGGCCGGTTACAAAATG
K13Q-Fwd	CATTGCAGGGCGAAACAACTAC
K13Q -Rev	CAACAAAGCGGCCTGTTACAAAATG

Supplementary Table 2 | Typical PCR reaction preparation

Component	Stock Solution	Volume Added (µl)
dNTPs	10 mM	1.0
Forward Primer	10 µM	2.5
Reverse Primer	10 µM	2.5
Template DNA	20 ng/µL	1.0
HF Buffer	5 X	
Ultrapure Water	-	32.5
Phusion DNA Polymerase	-	0.5
**Total Volume**	**-**	**50**

Supplementary Table 3 | Thermocycling conditions

Steps	Temperature	Time
Initial Denaturation	98℃	30 seconds
Denaturation	98℃	30 seconds
Primer Annealing (T_m)	54℃	20 seconds
Elongation	72℃	4 minutes
Final Elongation	72℃	10 minutes
Hold	4℃	-

Characterization of GB3 variants by 2D TOCSY-HSQC NMR.

^{1}H-^{15}N HSQC reveals the correlation of the backbone ^{15}N and its amide proton of each residue. TOCSY and NOESY spectra were sufficient to assign variants of WT GB3. As the example illustrated in Supplementary Fig. 3a, the amide proton NOE from the preceding T11 amide appears in the ^{N}H strip of L12, and the Hα NOE of L12 shows up in the strip for H13. A comparison of ^{1}H-^{15}N HSQC spectra of wt GB3, K13Q, and K13S is presented in Supplementary Fig. 3b, which shows K13 at 123.8 ppm, Q13 at 122.8 ppm, and S13 at 117.0 ppm.

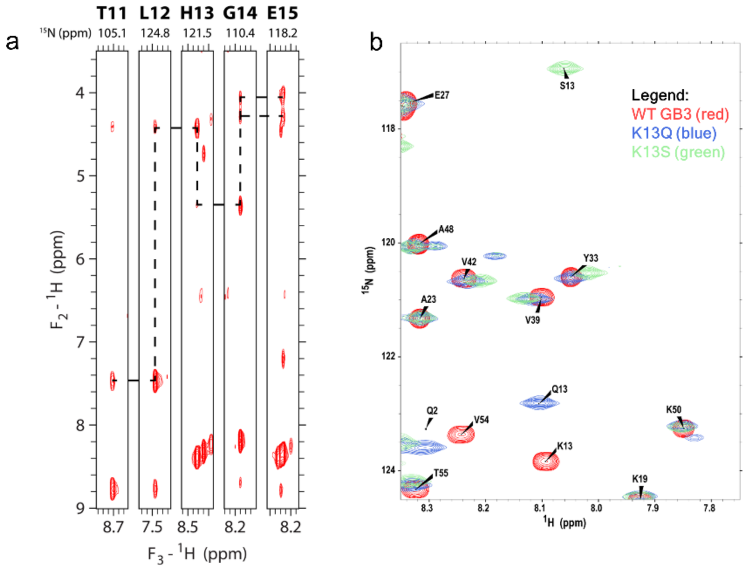

Supplementary Fig. 3 | Assignment of GB3 Variants. a, NOESY strip plot identifies assignment of residue 13 of K13H GB3 using the alpha proton connectivity. **b,** HSQC spectra comparing wt GB3 and two K13X variants. Differences in line widths result from differing acquisition times and do not reflect changes in protein motions.

Affinity scale for residue X quantified by 1D filtered NMR experiments

All α values are presented in Table 1, main text. The errors for all variants are identically represented by an average of 95% confidence intervals of 0.04 obtained from alpha values of 5 random variants with three observations. Each of these five variants were measured three times using independent replicate experiments. All other variants' alpha values were measured twice (Supplementary Table 4). The uncertainty for every GB3 variant should be identical, because all experiments are prepared and measured in the same way except for the variant of interest. The table below shows that the average 95% confidence interval of 0.04 is a reasonable upper limit for ascertaining alpha value differences. Moreover, all of the samples where two only two trials are performed are consistent with this confidence interval. The 95% confidence interval is calculated based on $\sigma = t * \frac{s}{\sqrt{n}}$ using the Excel built-in function of CONFIDENCE.T.

Supplementary Table 4 | Experimental replicates of alpha values and estimation of uncertainty

Variant	Alpha Trial 1	Alpha Trial 2	Alpha Trial 3	Sample Standard Deviation (*s*)	Average Alpha	95% CI
K13G	0.960	1.000		0.029	0.980	
K13A	0.652	0.668	0.661	0.008	0.660	0.019
K13L	0.432	0.368		0.045	0.400	
K13I	0.389	0.415		0.018	0.402	
K13V	0.445	0.462	0.459	0.009	0.455	0.022
K13M	0.723	0.764		0.029	0.743	
K13P	0.615	0.635		0.015	0.625	
K13F	0.448	0.474		0.019	0.461	
K13Y	0.463	0.470	0.489	0.014	0.474	0.034
K13W	0.401	0.423		0.016	0.412	
K13S	0.570	0.570		0.000	0.570	
K13C	5.847	5.536		0.220	5.692	
K13T	0.506	0.497		0.007	0.502	
K13E	0.374	0.356	0.324	0.025	0.352	0.063
K13D	0.488	0.445		0.030	0.466	
K13N	0.529	0.553		0.016	0.541	
K13Q	0.400	0.403		0.002	0.401	
K13H	0.816	0.766		0.035	0.791	
K13K	0.805	0.795	0.838	0.023	0.812	0.056
K13R	0.849	0.825		0.017	0.837	
				**Average Confidence Interval**		**0.04**

UV-vis titration for thermodynamic characterization of GB3 adsorption on AuNP

UV-vis titrations were performed on 5 selective GB3 variants with an Olis-refurbished Agilent 8453 spectrophotometer. A series of 15 titration samples were prepared by adding protein with increasing concentrations (0-1600 nM) into 2.0 nM AuNP solutions in 20 mM HEPES (pH 6.5). UV-vis measurement was conducted after 1 h sample incubation. Subsequently, the maximum AuNP plasmonic peak wavelength for each sample was interpolated to a precision of 0.05 nm, using polynomial fitting with an order of 8.

Surface prediction for AuNP binding using alpha values

First, the PDB file of a protein of interest (e.g., pepsin, 3PEP) is downloaded and opened with PyMOL (Schrodinger). Water molecules, extra chains, and hetero atoms are removed with PyMOL and the "clean" protein structure is saved as a new PDB file (`3pep_processed.pdb`). The calculation of binding affinity for each residue is completed by an in-house python script "asa_alpha_all-bfac.py" below. This script, and all others, are maintained at the Fitzkee Lab GitHub repository (https://github.com/FitzkeeLab/citrate-aunp-predict)[1]. Briefly, the binding affinity of each residue is calculated as a multiplication product of α values (ALPHA) and relative side chain accessible surface area (RASA). RASA was obtained by running NACCESS,[2] and only RASA larger than 25% was used. A new PDB file (`3pep_alpha.pdb`) is created with the B-factor column changed into product. The command used line is:

```
python3 ./asa-alpha_all_bfac.py 3pep_processed.pdb 3pep_alpha.pdb
```

To visualize the binding affinity with the use of "virtual atoms", we ran another in-house python script "binding_surface.py" on the modified PDB file (`3pep_alpha.pdb`) generated from

the last step. In this processing script, the protein is placed on a $1 \times 1 \times 1$ angstrom grid, and only the grid points that are more than two angstroms, but less than three angstroms from protein atoms are selected. This selects only the grid points on the surface and some cavities. A virtual "atom" is placed at each grid point. The B-factor of for each virtual atom is calculated as the average product ($\alpha \times RASA$) of all protein atoms within 10 angstroms of the grid point. Only the grid points with an average product greater than 30 are written in a new PDB file. The output will list the maximum b-factor and the x, y, z coordinate of the largest b-factor. The command line to run this script is:

```
python3 ./binding_surface.py 3pep_alpha.pdb 3pep_mesh.pdb
```

Finally, the structure can be visualized using `3pep_alpha.pdb` and `3pep_mesh.pdb` in PyMOL. The following commands to depict the average values from white to red, to indicating low vs. high binding affinity, as presented in the text.

```
show spheres, 3pep_mesh
spectrum b, white_red, 3pep_mesh, minimum=30, maximum=50,
selection=3pep_mesh
set sphere_scale, 0.5, 3pep_mesh
```

Additional instructions are included on the GitHub site.

Proteinase K (PK) binding capacity determination

In order to control for the concentration of AuNP-bound PK in the proteolytic reaction, the binding capacity of PK on 15-nm AuNP must be quantified. Determining the binding capacity allows for the stoichiometry to be precisely controlled during activity measurements. We use 1D proton NMR to determine the binding capacity on 15 nm AuNPs[3]. Briefly, 50 nM of AuNP was mixed with 20 µM PK in 10 mM KH_2PO_4 at pH 7.5, the proton intensities of which are compared with those from 20 µM PK without addition of AuNPs. A scale factor (0.795 ± 0.014; uncertainty is given as the standard deviation of ≥ 3 independent replicate samples) was obtained using TOPSPIN, which corresponds to the unbound fraction of PK. The binding capacity of PK (C_{PK}) was determined as 82 ± 6 PK per AuNP (Supplementary Fig. 4).

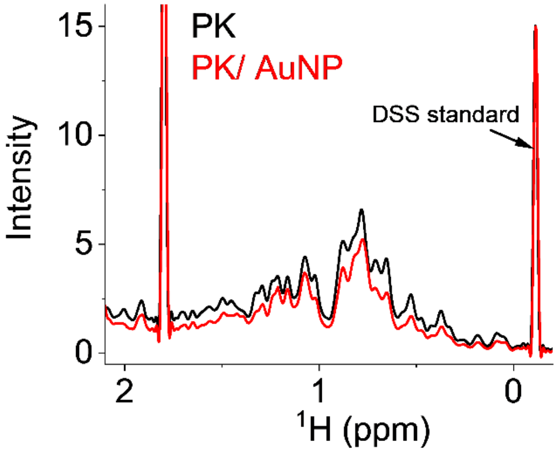

Supplementary Fig. 4 | 1D proton NMR spectra of 20 µM PK sample (black, PK) and 20 µM PK mixed with 50 nM AuNP (red, PK/AuNP). The signal is calibrated with DSS standard in each spectrum, and signal reduction is caused by the bound PK, with which the binding capacity C_{PK} was calculated.

Human carbonic anhydrase (HCA) binding capacity determination

The binding capacity of HCA towards AuNPs was quantified by 1D NMR identically as for PK, and it was found to be 42 ± 8 HCA per NP (uncertainty is given as the standard deviation for ≥ 3 independent replicate samples, representative data shown in Supplementary Fig. 5), and the two AuNP-bound HCA samples (in-situ and purified) were prepared in the same way, except that in the in-situ method 100% HCA was bound to AuNPs.

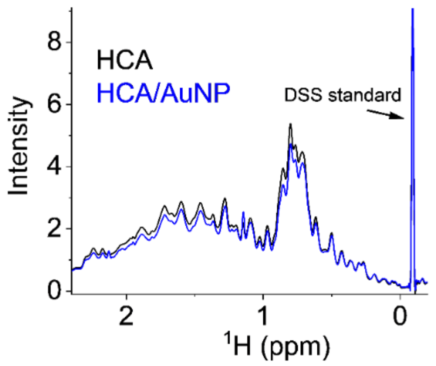

Supplementary Fig. 5 | 1D proton NMR spectra of 20 µM HCA sample (black, HCA) and 20 µM HCA mixed with 50 nM AuNP (blue, HCA/AuNP). The signal is calibrated with DSS standard in each spectrum, and signal reduction corresponds to bound HCA concentration.

Proteinase K proteolytic activity assay with 10 nm and 30 nm AuNPs

To determine the surface curvature's effect on the PK's orientation, the PK proteolytic activity assay was repeated with 10 nm and 30 nm AuNP (AuNP@PK). The samples were prepared as described in the main text with the following modifications to account for the 10 nm and 30 nm AuNP surface area. The binding capacity of PK was determined for 10 nm and 30 nm AuNP from 1D proton NMR[4]. The proton intensities of 20 µM PK mixed with 28 nM (10 nm) or 40 nM (30 nm) AuNP were compared with 20 µM free PK in potassium phosphate buffer, pH 7.2. The binding capacity of PK was determined to be 72 and 200 PK per AuNP for 10 and 30 nm AuNP, respectively.

For the in-situ method, 0.01 mg/mL PK was incubated with 5 nM AuNP (10 nm), and 0.03 mg/mL PK was incubated with 5 nM AuNP (30 nm) to saturate all the binding surface of AuNP. For the washing method, 0.06 mg/mL PK was incubated with 5 nM AuNP (10 nm) and 0.12 mg/mL PK was incubated with 5 nM AuNP (30 nm). Samples with AuNP were incubated for three hours before washing off excess PK from the samples, which was performed by centrifugation at 9,000 *g* for 15 min. The washing step was repeated three times, and the concentration of AuNP@PK was determined before the PK activity assay.

Finally, the PK activity assay for AuNP(10)@PK was done with either 0.01 mg/mL free PK, in-situ AuNP@PK, or purified AuNP@PK and 2 mg/mL BSA at room temperature. Similarly, PK activity assay for AuNP(30)@PK was done with either 0.03 mg/mL free PK, in-situ AuNP@PK, or purified AuNP@PK and 2 mg/mL BSA. Samples for loading onto SDS-PAGE were prepared as described in the main text. Under these conditions, a partial digestion of 2 mg/mL BSA was observed for the in-situ reactions for 15 nm AuNP@PK..

HCA activity assay on 10 nm and 30 nm AuNPs

The HCA activity assay was done for 10 nm and 30 nm AuNP (AuNP@HCA) as described in the main text with the following modification to account for the 10 nm and 30 nm AuNP surface area. The binding capacity of HCA on 10 nm and 30 nm AuNP was determined as follows[4]: 18 μM HCA was mixed with 25 nM of either 10 nm or 30 nm AuNP and compared to a reference sample with 18 μM free HCA. The ratio of 1D NMR peak intensities in the amide proton region was used to estimate the fraction bound. The buffer for these experiments was 10 mM HEPES, pH 7.5. The binding capacity of HCA was determined to be 47 and 133 HCA per AuNP for 10 nm and 30 nm AuNP, respectively.

For in-situ enzyme activity experiments, 0.16 μM was incubated with 10 nM 10 nm AuNP, and 1.1 μM HCA was incubated with 10 nM 30 nm AuNP. The different concentrations allowed the protein to

saturate the binding surface of AuNPs, which is greater for 30 nm than 10 nm AuNPs, and the saturation stoichiometries were derived from the binding capacity experiments described above. For the washing method, 1.6 μM and 5 μM HCA was incubated with 10 nM AuNP (either 10 nm or 30 nm in size). Samples with AuNP were incubated for three hours before washing, which was performed by centrifugation at 9,000 *g* for 15 min, followed by resuspension in buffer. The washing step was repeated three times, and the concentration of AuNP@HCA was determined before the HCA activity assay.

Finally, the in-situ and purified HCA@AuNP activity samples were made using either 6 nM AuNP(10)@HCA or 6 nM AuNP(30)@HCA and 100 μM *p*NPA. A control HCA activity reaction was run using either 0.16 μM HCA or 1.1 μM and 100 μM *p*NPA. The activity assay was done as described in the main text.

Quantification of Charge Effect on GB3 binding onto AuNPs

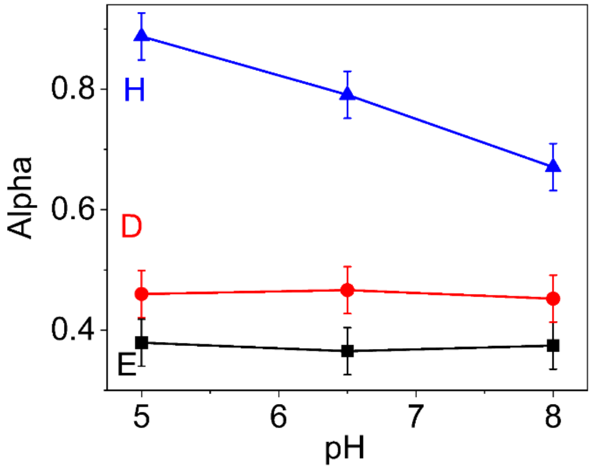

Supplementary Fig. 6 | Effect of pH change (from 5-8) on GB3 competitive binding with AuNPs. The blue, red and black data points represent K13H, K13D and K13E GB3 variants. Error bars are given as the standard error of the mean for n = 3 independent experiments.

Kinetics study of GB3 binding onto AuNPs with SOFAST-HMQC

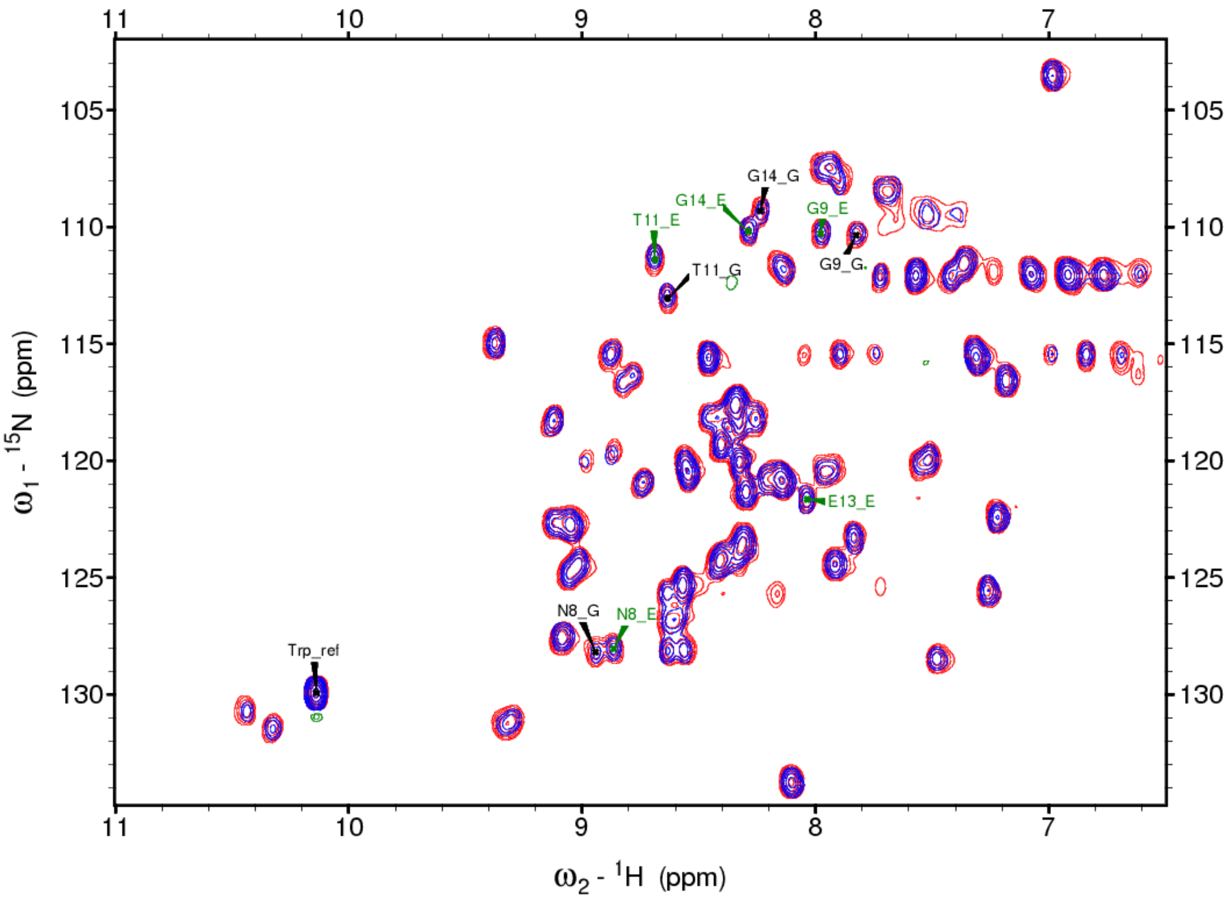

Supplementary Fig. 7 | Complete SOFAST-HMQC spectra of ^{15}N K13E/^{15}N K13G mixture without (red) and with (blue) AuNPs after incubation of 365 mins. The signal reduction for the blue spectrum as compared to the red one is due to protein adsorption onto AuNPs. Well-resolved peak assignments for K13G ("_G") and K13E ("_E") are used for quantitative analysis. Because the proteins only differ by one residue, the majority of peaks overlap.

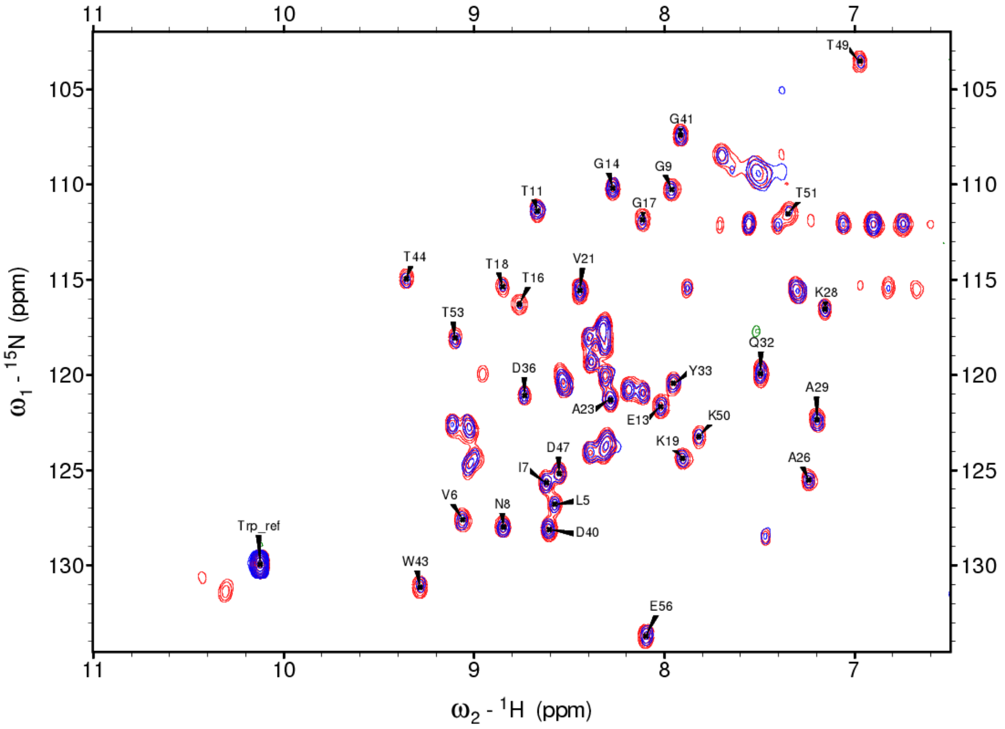

Supplementary Fig. 8 | Representative SOFAST-HMQC spectra of ^{15}N K13E without (red) and with (blue) AuNPs for 365 mins. The intensity of Trp reference is used to calibrate residue peak intensities. The assigned peaks are well resolved and used for quantitative analysis.

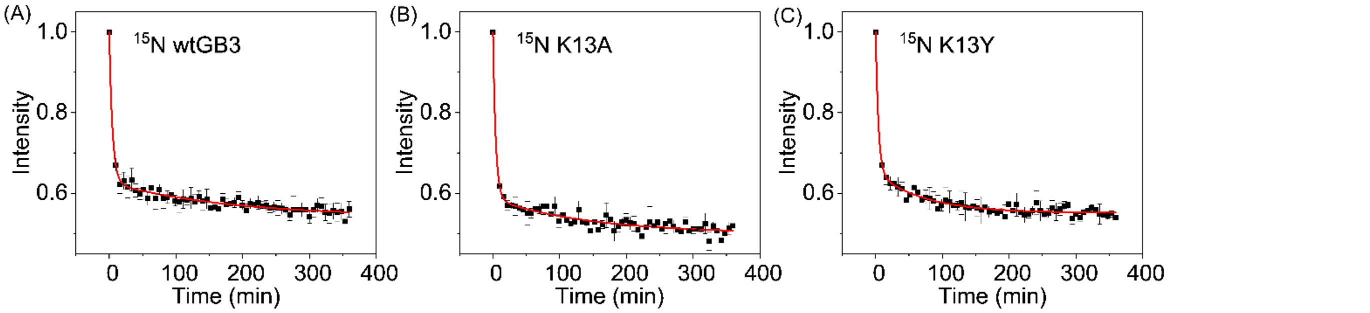

Supplementary Fig. 9 | Kinetics Measurements for GB3 Variants. Normalized average peak intensities of 20 µM of (A) ^{15}N wt (B) ^{15}N K13A, and (C) ^{15}N K13Y as a function of incubation time after mixing with 50 nM AuNPs. The 0 min intensity is acquired with protein samples without AuNPs, by which all peak intensities are normalized. The data presented here are representative, and two independent kinetics measurements were performed for each variant to ensure reproducibility. Error bars represent the standard error of the mean of n ≈ 50 resolved peak intensities from a single experiment.

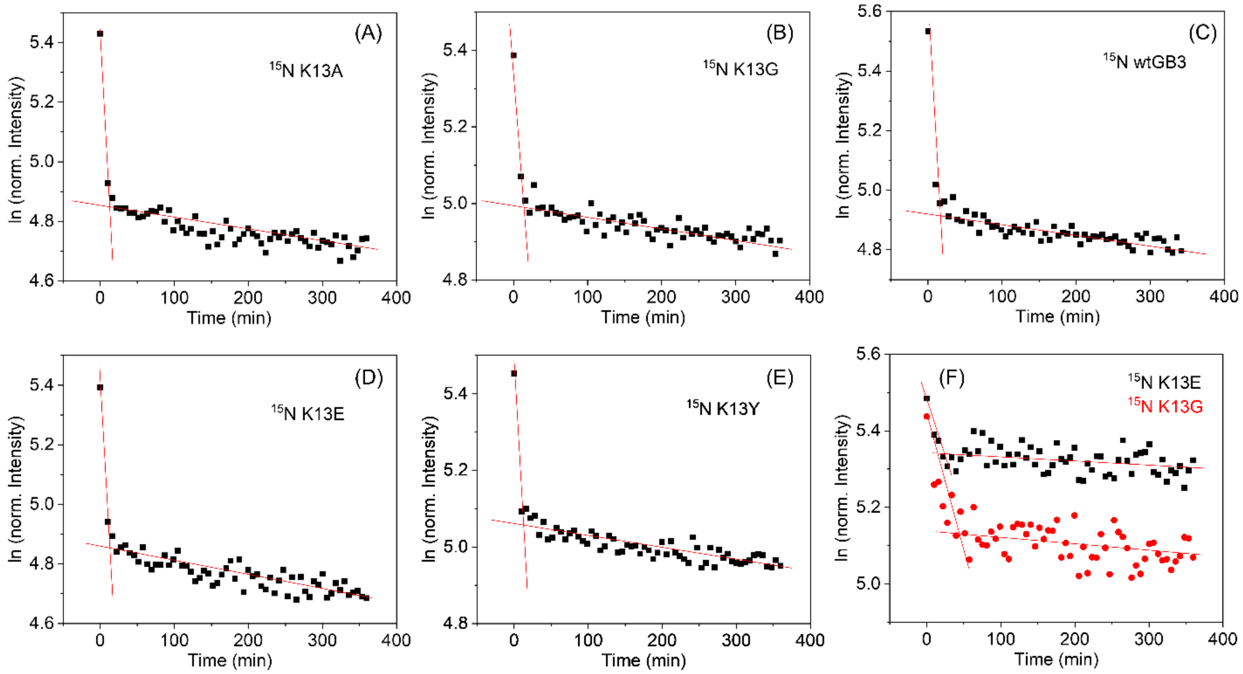

Supplementary Fig. 10 | Semi-log plot of normalized peak intensities of GB3 variants as a function of time in kinetic binding study. The data are identical to what is shown in **Supplementary Fig. 9** and **Fig. 3**, and red lines show the pseudo-first order fit. **(A)-(E)** are from the individual binding of five selected GB3 variants, and **(F)** is from the competitive binding of mixture of K13E and K13G. The normalized peak intensities are derived by normalizing protein peak intensities by the ^{15}N-Trp reference signal and multiplied by a factor of 1000. No evidence for additional exponential timescales is observed.

Supplementary Table 5 | Kinetics time constants of GB3 variants using two-process decay model

Parameter	K13Y	K13A	wtGB3	K13G	K13E	K13E-mixture	K13G-mixture
**y_0**	0.55±0.01	0.50±0.01	0.54±0.01	0.50±0.01	0.50±0.01	0.86±0.01	0.70±0.01
**A_1**	0.35±0.01	0.41±0.01	0.37±0.01	0.39±0.01	0.40±0.01	0.13±0.07	0.23±0.02
**t_1 (min)**	**4.2±0.6**	**4.2±0.6**	**4.8±0.5**	**4.1±0.5**	**4.8±0.5**	**9±7**	**10±2**
**A_2**	0.10±0.01	0.08±0.01	0.08±0.01	0.10±0.01	0.09±0.01	0.01±0.07	0.07±0.01
**t_2 (min)**	70±10	150±50	180±50	90±14	140±30	50±400	200±50
**R^2**	0.98	0.97	0.98	0.98	0.97	0.46	0.82

The kinetic data are fitted with equation $y = y_0 + A_1 e^{(-\frac{x}{t_1})} + A_2 e^{(-\frac{x}{t_2})}$, where x refers to incubation time in mins and y to normalized residue intensities. Only the pseudo first-order time (t_1 and t_2) reflect the kinetics of binding. The errors in Table 5 represent fitting errors and may not reflect experimental uncertainties from repeated measurements.

Characterization of Purified AuNP@PK

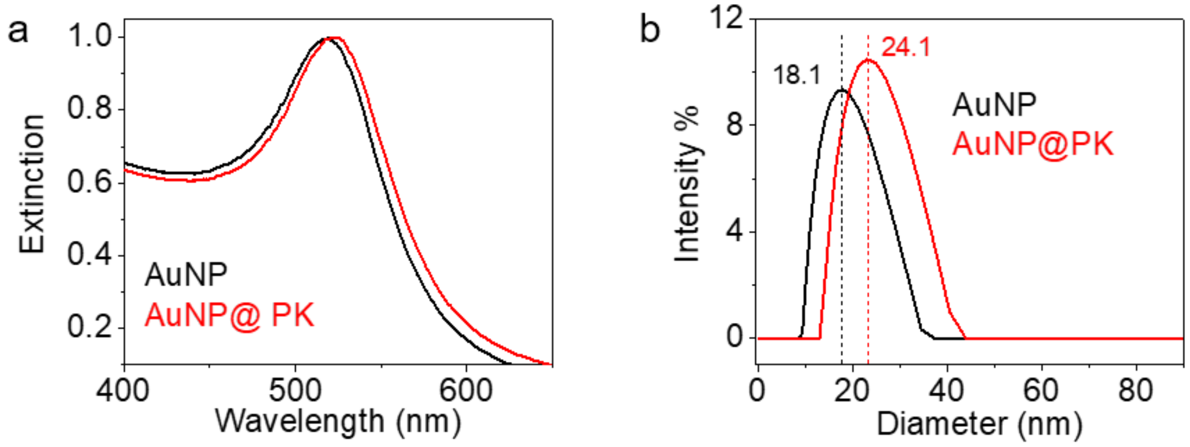

Supplementary Fig. 11 | UV-Vis and DLS Data for AuNP@PK. a, UV-vis spectra, and b, DLS analysis of the purified pre-coated AuNP@PK after washing. The red shift of peak extinction wavelength of AuNP in UV-vis and increased hydrodynamic diameter of AuNP are caused by the adsorption of PK.

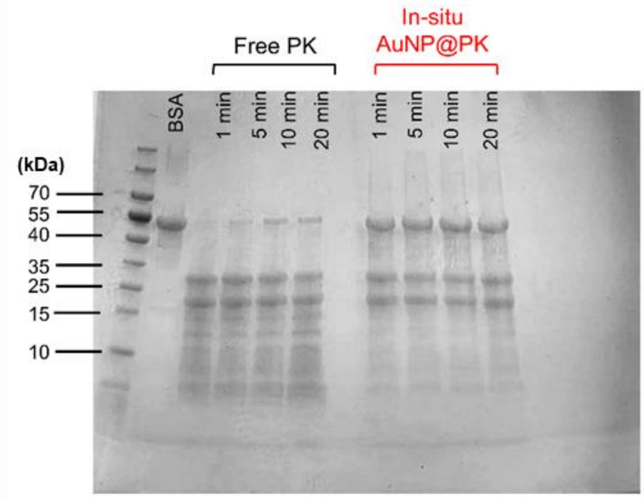

Supplementary Fig. 12 | AuNP-bound BSA is protected from proteolysis by PK when excess AuNP is added in preparation of in-situ AuNP@PK. With the binding capacity determined with 1D NMR, 6.8 nM AuNP is required to fully bind 0.01 mg/mL PK. Here, we incubated 0.01 mg/mL PK with 30 nM AuNP (> 4 times in excess) before adding BSA. In contrast to the results presented in the main text where AuNP is not in excess, a significant fraction of AuNP-bound BSA is not cleaved by PK, and the digestion is incomplete.

Characterization of purified AuNP@HCA

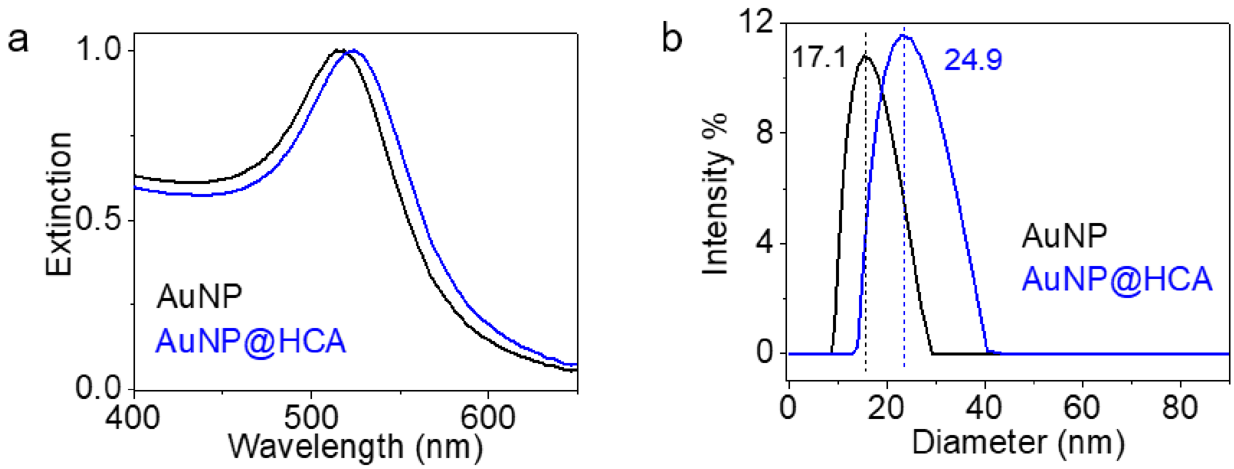

Supplementary Fig. 13 | a, UV-vis spectra, and **b,** DLS analysis of the purified pre-coated AuNP@HCA after washing. The red shift of peak extinction wavelength of AuNP in UV-vis and increased hydrodynamic diameter of AuNP are caused by the binding of HCA.

Examination of Enzyme Activities on 10 nm and 30 nm AuNPs

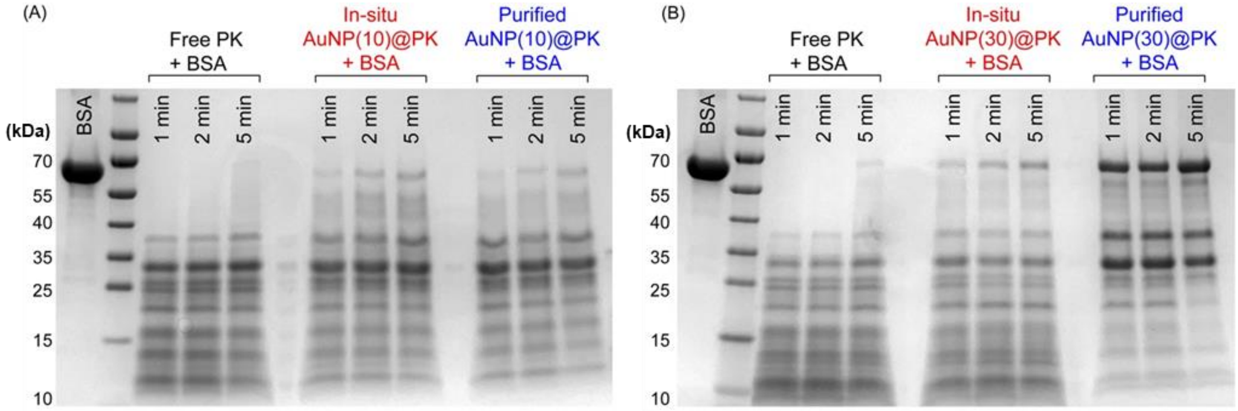

Supplementary Fig. 14 | Effect of AuNP surface curvature on the bound orientation of PK. PK activity assay with 10 nm **(A)** and 30 nm AuNP-bound PK **(B)**. The PK concentration in assays is kept at 0.01 and 0.03 mg/mL for 10 and 30 nm AuNP, respectively. A 2 mg/mL BSA substrate concentration is used for all samples. The free, in-situ, and purified AuNP@PK samples are prepared as described in the main text for 15 nm AuNP@PK (Fig 4). For both 10 and 30 nm AuNP@PK, the SDS-PAGE shows limited proteolysis of BSA using free PK (lane 3-5), in-situ AuNP@PK (lane 7-9), and pre-coated and washed AuNP@PK (lane 11-13) for reaction times of 1 min, 2 mins, and 5 mins (from left to right), respectively. Lane 1 shows a 2 mg/mL BSA control with no PK.

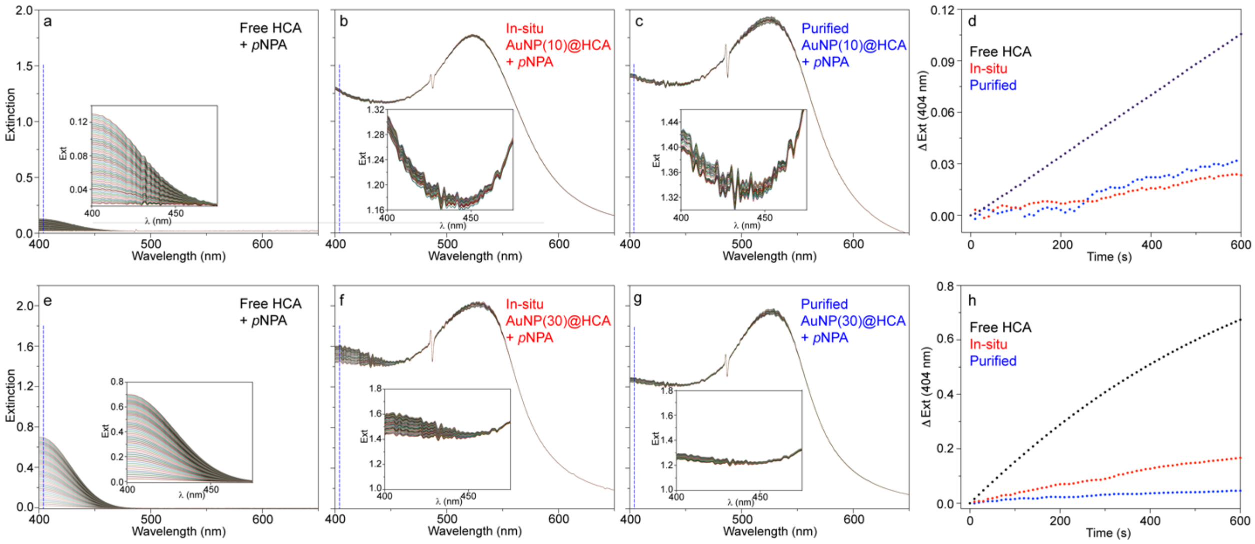

Supplementary Fig. 15 | Effect of AuNP surface curvature on the bound orientation of HCA. HCA activity assay with 10 nm **(a-d)** and 30 nm AuNP-bound HCA **(e-h)**. The enzyme/substrate ratio is fixed at 0.16 μM HCA/100 μM *p*NPA and 1.1 μM HCA/100 μM *p*NPA for 10 and 30 nm AuNP@PK, respectively. Time-resolved UV-vis spectra of HCA assay solutions with an incubation time of 10 mins using free HCA **(a, e)** in-situ HCA bound to AuNPs (AuNP@HCA, **b, f**), and washed/purified AuNP@HCA **(c, g)**. Close-up UV-Vis spectra are shown as insets in **a-c** and **e-g**. Comparisons of extinction change at 404 nm as a function of reaction time using free HCA (black), in-situ AuNP@HCA (red), and purified AuNP@HCA (blue) are shown for 10 nm **(d)** and 30 nm AuNP@HCA **(h).**

Predicted Interaction Surface of Human Fibrinogen

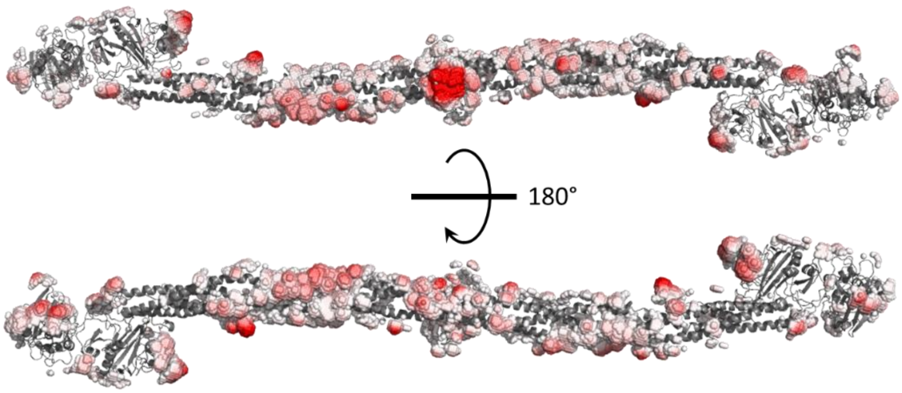

Supplementary Fig. 16 | Predicted binding surface of human fibrinogen. The front and rear faces of fibrinogen (PDB 3GHG) overlaid with the mesh of average weighted alpha values is shown. The long coiled-coil region is favored for binding, suggesting that the long axis would be preferred for binding to an AuNP surface. Previous work by Roach et al. suggests that binding of fibrinogen to surfaces occurs in multiple steps, where binding along the long axis occurs first, followed by rearrangement to end-on binding[5]. This complex behavior is not predicted by the alpha value, which does not account for potential rearrangement after adsorption.

Comparison with Computational Simulations of Protein-AuNP Binding

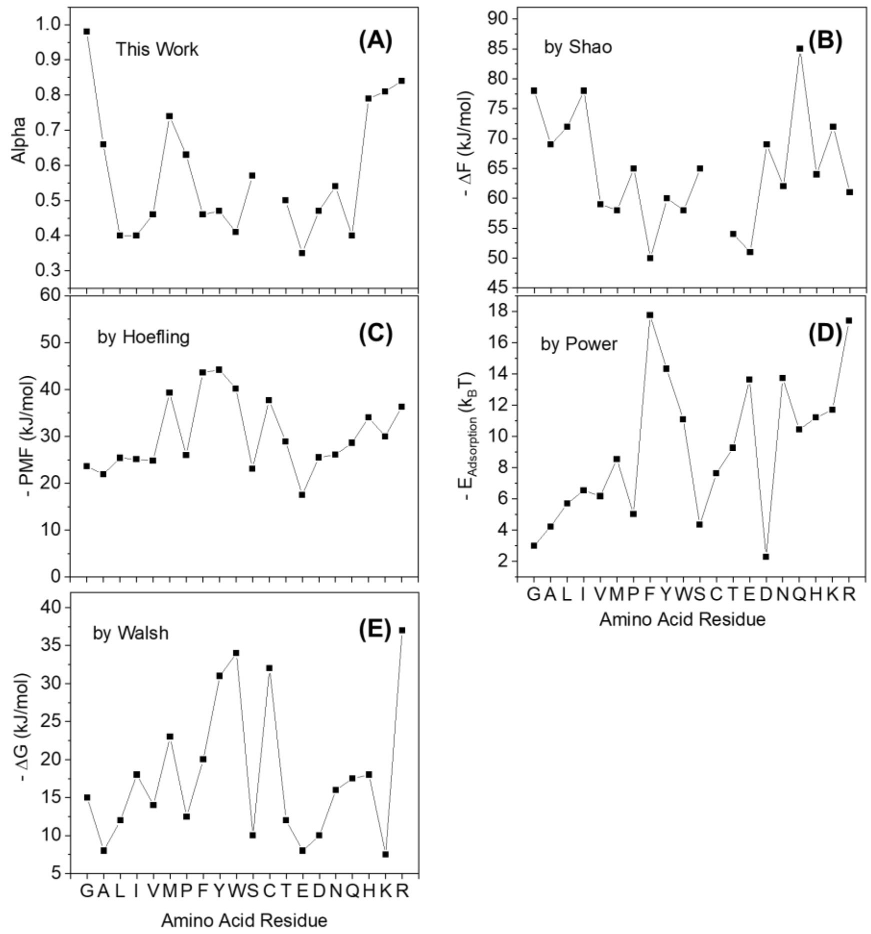

Supplementary Figure 17. | Comparison of Alpha Values to Other Residue Scales. A comparison between alpha values in this work **(A)** and amino acid-nanoparticle binding energies **(B-E),** where binding energies were determined by computational simulations. Results are shown for Shao and Hall **(B)**[6]; Hoefling *et al.*, **(C)**[7,8]; Power *et al.*, **(D)**[9]; and Walsh, **(E)**[10,11]. Since the binding energies are favorable (negative), we have negated the original values for comparison to alpha values. On all graphs above, larger values reflect more a favorable interaction in the system studied. Because of the magnitude of the Cys alpha value, this point is omitted from **(A)**.

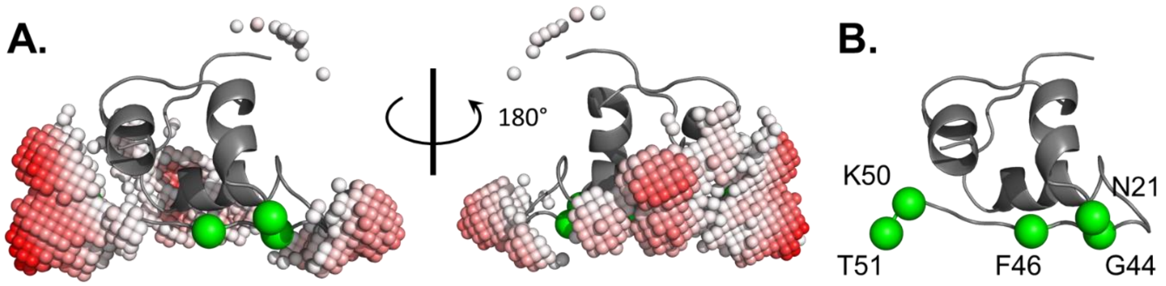

Supplementary Figure 18. | A Comparison of the Alpha-Predicted Insulin Binding Orientation with Molecular Dynamics Simulations. (A) Alpha-value predicted binding surface for human insulin (PDB 4EWZ). For this calculation, Cys residues were assigned an intermediate alpha value of 0.5 because all Cys residues in insulin form disulfides and are not accessible to bind gold. **(B)** The same insulin orientation shown in (A), without the alpha-value predicted surface. The C_α atoms of residues with significant citrate-AuNP binding propensity according to simulations[12] are shown as green spheres. These spheres are labeled according to the numbering scheme of Tavanti, *et al*.[12].

Survey of Literature-Reported AuNP-bound Enzyme Activities

**Enzyme 1**	**Alcohol dehydrogenase (TbADH)**
PDB ID	1YKF
NP properties	15-nm-Mercaptopropionic acid-capped AuNP
AuNP-bound activity from literature	Activity decreases from 12.5 U to 3.5U when bound.[13]
Active site(s)	Residues 37, 59, 150 for Zn^{2+} binding, and residues 218, 340 for NADP binding.[14]
Analysis	Active sites should experience some steric hindrance from AuNP, but will not be completely blocked.

Predicted Binding Surface:

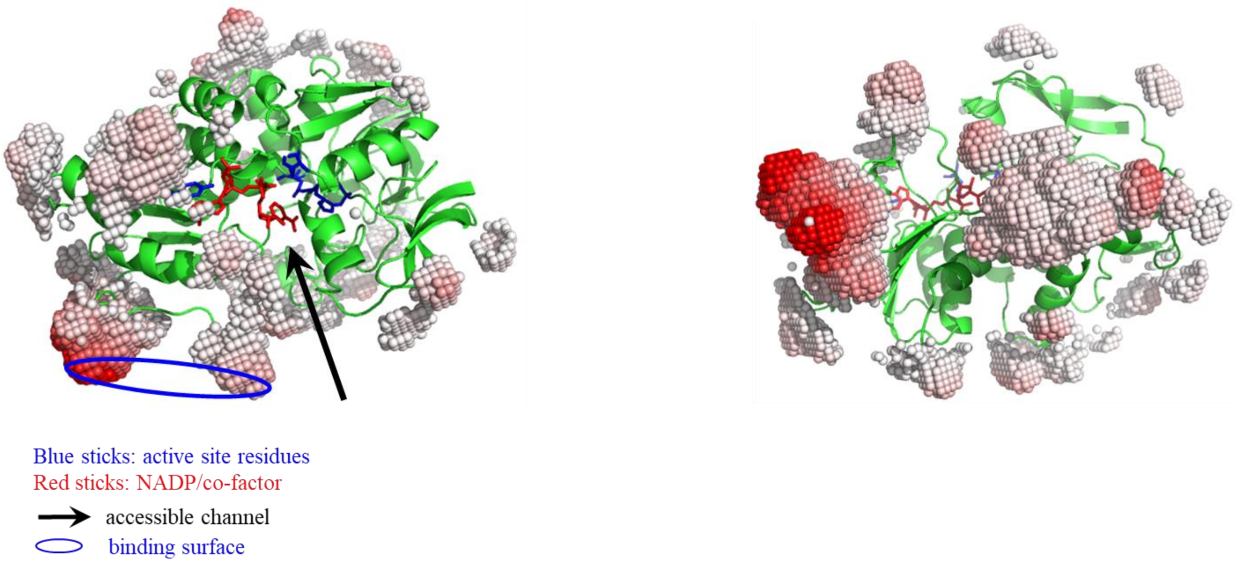

Enzyme 2	Acetylcholinesterase (AChE)
PDB ID	1EEA
NP properties	14 nm citrate capped AuNP
AuNP-bound activity from literature	Activity is fully retained.[15]
Active site(s)	Residues Ser 200, His 440, and Glu 327 form a catalytic triad.[16]
Analysis	The channel to the catalytic triad should not be blocked.

Predicted Binding Surface:

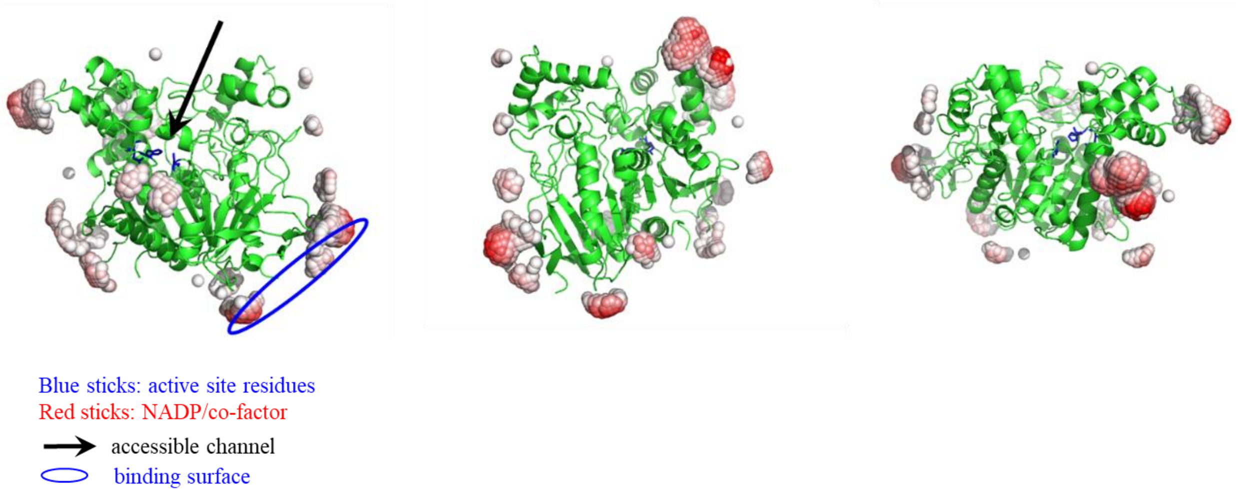

Enzyme 3	Citrate synthase (CS)
PDB ID	1CTS
NP properties	14 nm citrate capped AuNP
AuNP-bound activity from literature	Activity is fully retained.[15]
Active site(s)	His 274, His 320, and Asp 375[17]
Analysis	The channel to the catalytic triad should not be blocked.

Predicted Binding Surface:

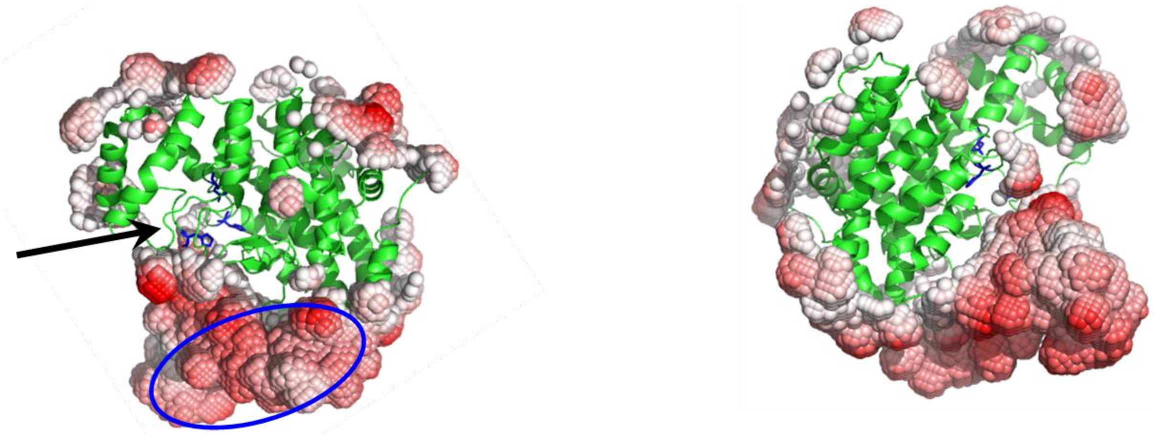

Blue sticks: active site residues

accessible channel

binding surface

Enzyme 4	Horseradish peroxidase (HRP)
PDB ID	1HCH
NP properties	14 nm citrate-capped AuNP
AuNP-bound activity from literature	Activity is only 50% retained, and further decreases to 5% when HRP packing density increases[18]
Active site(s)	A hydrophobic pocket formed by His 42, Phe 68, Gly 69, Ala 140, Pro 141, Phe 142, and Phe 179[19]
Analysis	The access to the heme and active site should be sterically hindered. Increasing packing density may enhance hindrance from nearby HRP, which decreases activity but not considered by our model.

Predicted Binding Surface:

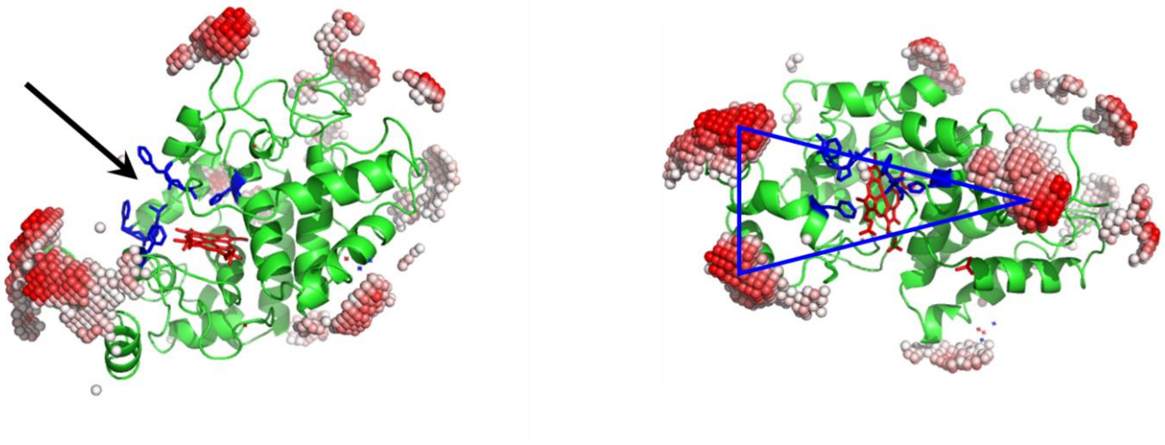

Blue sticks: active site residues
Red sticks: heme
→ accessible channel
⬭ binding surface

Enzyme 5	Cytochrome *c* (Cyt C)
PDB ID	1OCD
NP properties	10-nm citrate-capped silver NP (AgNP)
Bound orientation from literature	The heme ring plane lies at a slight angle to the NP surface at low coverage. It re-orients to a more vertical orientation at high coverage.[20]
Analysis	Cyt *c* should bind at the back (blue circle), where the highest binding affinity region lies. This will orient the heme ring towards AgNP, which is consistent with observations. However, Cyt *c* has several regions of high affinity binding patches, which may explain its reorientation.

Predicted Binding Surface:

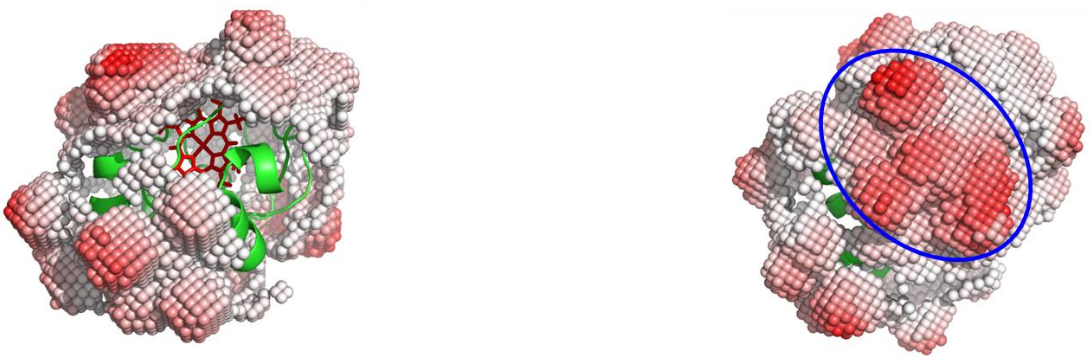

Blue sticks: active site residues
Red sticks: heme
→ accessible channel
⬭ binding surface

Enzyme 6	Glucose Oxidase (GOx)
PDB ID	1GAL
NP properties	10-nm citrate-capped AuNP
AuNP-bound activity from literature	Activity decreases by half. Authors attributed the loss of function to protein unfolding on NP.[21]
Active site(s)	His 516, Glu 412, and His 559[22]
Analysis	Our model predicts no steric hindrance and does not explain the observed result.

Predicted Binding Surface:

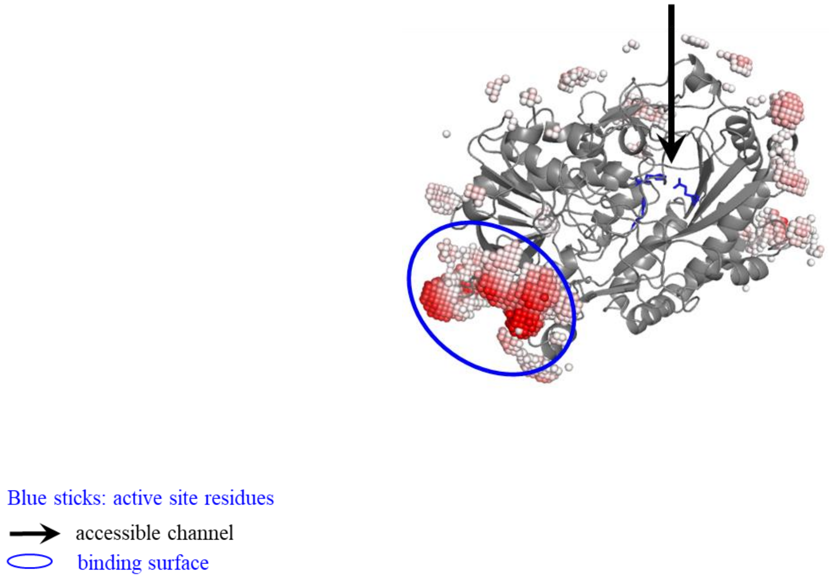

^{1}H and ^{15}N Chemical Shifts of 20 GB3 variants

Supplementary Table 6. Chemical Shifts of WT GB3, K13H, and K13A GB3 variants

Residues in WT GB3		WT GB3 ^{15}N (ppm)	WT GB3 ^{1}H (ppm)	K13H GB3 ^{15}N (ppm)	K13H GB3 ^{1}H (ppm)	K13A GB3 ^{15}N (ppm)	K13A GB3 ^{1}H (ppm)
2	Gln	123.27	8.31	123.54	8.35	124.02	8.39
3	Tyr	124.31	9.04	124.32	9.04	124.33	9.04
4	Lys	122.85	9.09	122.71	9.10	122.58	9.07
5	Leu	126.82	8.61	126.66	8.61	126.79	8.64
6	Val	127.32	9.15	127.12	9.10	127.49	9.11
7	Ile	125.73	8.76	125.50	8.69	125.61	8.67
8	Asn	129.35	8.98	129.20	8.92	128.29	8.91
9	Gly	110.52	7.91	110.29	7.83	110.15	7.80
10	Lys	120.97	9.52	120.24	9.36	120.03	9.12
11	Thr	109.12	8.76	109.16	8.77	111.51	8.72
12	Leu	125.95	7.57	124.85	7.47	125.12	7.76
13	Lys	123.84	8.09	121.60	8.39	124.12	7.95
14	Gly	109.46	8.30	110.45	8.19	108.50	8.24
15	Glu	118.33	8.38	118.28	8.35	118.56	8.35
16	Thr	115.83	8.81	115.92	8.77	116.51	8.84
17	Thr	111.99	8.11	111.96	8.12	111.65	8.15
18	Thr	115.28	8.91	115.29	8.92	115.44	8.91
19	Lys	124.53	7.93	124.49	7.93	124.48	7.93
20	Ala	124.95	9.08	124.84	9.09	124.92	9.09
21	Val	115.46	8.46	115.71	8.49	115.56	8.46
22	Asp	115.58	7.32	115.56	7.31	115.58	7.32
23	Ala	121.33	8.32	121.34	8.31	121.32	8.31
24	Glu	119.28	8.42	119.23	8.41	119.29	8.41
25	Thr	117.60	8.34	117.62	8.34	117.79	8.36
26	Ala	125.47	7.23	125.46	7.24	125.52	7.26
27	Glu	117.60	8.34	117.55	8.34	117.41	8.36
28	Lys	116.55	7.17	116.59	7.17	116.57	7.19
29	Ala	122.39	7.21	122.41	7.19	122.43	7.23
30	Phe	119.97	8.58	119.93	8.58	120.02	8.58
31	Lys	123.13	9.01	123.16	9.01	122.91	9.06
32	Gln	119.80	7.48	119.80	7.47	120.00	7.54
33	Tyr	120.60	8.05	120.61	8.04	120.47	7.98
34	Ala	122.69	9.18	122.74	9.20	122.64	9.13
35	Asn	118.18	8.37	118.19	8.37	118.11	8.45

Residues in WT GB3		WT GB3 ^{15}N (ppm)	WT GB3 ^{1}H (ppm)	K13H GB3 ^{15}N (ppm)	K13H GB3 ^{1}H (ppm)	K13A GB3 ^{15}N (ppm)	K13A GB3 ^{1}H (ppm)
36	Asp	121.45	8.82	121.47	8.79	121.10	8.79
37	Gln	115.53	7.37	115.58	7.37	115.23	7.33
38	Gly	108.38	7.78	108.43	7.78	108.44	7.72
39	Val	120.95	8.10	120.87	8.09	120.94	8.14
40	Asp	128.301	8.723	128.078	8.675	128.331	8.676
41	Gly	107.470	7.896	107.516	7.930	107.322	7.949
42	Val	120.624	8.238	120.714	8.219	120.607	8.215
43	Trp	131.294	9.308	131.342	9.323	131.210	9.314
44	Thr	114.763	9.371	114.909	9.393	114.911	9.383
45	Tyr	120.386	8.551	120.490	8.564	120.537	8.554
46	Asp	128.541	7.560	128.515	7.553	128.498	7.510
47	Asp	125.161	8.571	125.149	8.577	125.146	8.572
48	Ala	120.024	8.319	120.068	8.324	120.071	8.327
49	Thr	103.394	6.987	135.658	6.995	135.687	6.994
50	Lys	123.258	7.849	123.254	7.851	123.273	7.847
51	Thr	111.486	7.392	111.424	7.388	111.410	7.369
52	Phe	131.383	10.363	131.400	10.371	131.497	10.351
53	Thr	117.798	9.145	117.986	9.149	118.099	9.136
54	Val	123.362	8.242	123.680	8.289	123.380	8.334
55	Thr	124.326	8.325	124.370	8.342	124.221	8.353
56	Glu	133.835	7.867	133.952	7.914	133.926	8.058

Supplementary Table 7. ^{1}H and ^{15}N Chemical Shift values of K13F, K13N, and K13Y GB3 variants

Residues in WT GB3		K13F GB3 ^{15}N (ppm)	K13F GB3 ^{1}H (ppm)	K13N GB3 ^{15}N (ppm)	K13N GB3 ^{1}H (ppm)	K13Y GB3 ^{15}N (ppm)	K13Y GB3 ^{1}H (ppm)
2	Gln	123.29	8.33	123.34	8.33	-	-
3	Tyr	124.30	9.03	124.33	9.05	124.29	9.04
4	Lys	122.60	9.08	122.66	9.08	122.72	9.10
5	Leu	126.63	8.58	126.82	8.62	126.70	8.57
6	Val	127.29	9.04	127.40	9.09	127.11	9.00
7	Ile	125.75	8.65	125.58	8.66	125.85	8.67
8	Asn	127.76	8.86	128.44	8.87	126.92	8.80
9	Gly	110.25	7.91	110.67	7.98	113.00	7.88
10	Lys	120.21	9.23	120.51	9.30	120.87	9.35
11	Thr	109.50	8.76	110.01	8.80	108.86	8.80
12	Leu	124.80	7.45	124.72	7.53	124.31	7.38
13	Lys	122.31	7.98	121.18	8.21	123.32	8.05
14	Gly	111.31	8.05	109.31	8.33	110.47	7.99
15	Glu	118.12	8.23	118.38	8.36	117.27	8.13
16	Thr	116.25	8.74	116.15	8.82	115.94	8.69
17	Thr	112.00	8.12	111.90	8.12	111.93	8.11
18	Thr	115.36	8.90	115.37	8.91	115.31	8.91
19	Lys	124.52	7.92	124.55	7.93	124.60	7.93
20	Ala	124.90	9.08	124.93	9.09	124.94	9.07
21	Val	115.61	8.47	115.54	8.47	115.50	8.45
22	Asp	115.61	7.32	115.57	7.32	115.56	7.31
23	Ala	121.33	8.31	121.33	8.32	121.35	8.32
24	Glu	119.26	8.40	119.30	8.41	119.24	8.40
25	Thr	117.40	8.34	117.76	8.36	117.64	8.33
26	Ala	125.48	7.24	125.50	7.26	125.48	7.22
27	Glu	117.71	8.34	117.42	8.36	117.64	8.33
28	Lys	116.55	7.17	116.59	7.18	116.53	7.16
29	Ala	122.40	7.21	122.43	7.22	122.39	7.19
30	Phe	119.94	8.57	120.00	8.59	119.94	8.57
31	Lys	123.06	9.02	123.11	9.05	123.15	9.01
32	Gln	119.82	7.49	119.92	7.51	119.76	7.46
33	Tyr	120.56	8.02	120.60	8.03	120.65	8.03
34	Ala	122.71	9.17	122.73	9.18	122.72	9.18
35	Asn	118.12	8.38	118.16	8.40	118.12	8.34
36	Asp	121.39	8.78	121.34	8.80	121.46	8.77
37	Gln	115.53	7.36	115.42	7.36	115.61	7.38

Residues in WT GB3		K13F GB3 ^{15}N (ppm)	^{1}H (ppm)	K13N GB3 ^{15}N (ppm)	^{1}H (ppm)	K13Y GB3 ^{15}N (ppm)	^{1}H (ppm)
38	Gly	108.47	7.77	108.57	7.77	108.59	7.78
39	Val	120.90	8.09	121.00	8.13	120.78	8.06
40	Asp	128.05	8.66	128.43	8.74	127.74	8.61
41	Gly	107.47	7.94	107.53	7.93	107.50	7.95
42	Val	120.76	8.21	120.63	8.22	120.86	8.17
43	Trp	131.26	9.31	131.13	9.30	131.38	9.32
44	Thr	114.94	9.39	114.87	9.39	115.02	9.42
45	Tyr	120.54	8.55	120.50	8.55	120.56	8.55
46	Asp	128.51	7.53	128.53	7.53	128.50	7.53
47	Asp	125.12	8.57	125.13	8.57	125.10	8.56
48	Ala	120.05	8.32	120.06	8.33	120.06	8.32
49	Thr	135.69	7.00	135.67	7.00	135.70	6.99
50	Lys	123.21	7.85	123.23	7.85	123.17	7.84
51	Thr	111.41	7.38	111.43	7.38	111.35	7.36
52	Phe	131.42	10.35	131.44	10.37	131.48	10.35
53	Thr	117.95	9.13	117.94	9.13	118.05	9.14
54	Val	123.92	8.34	124.05	8.31	124.00	8.39
55	Thr	124.21	8.35	123.87	8.38	124.24	8.36
56	Glu	134.01	8.00	133.87	8.08	134.28	7.93

Supplementary Table 8. ^{1}H and ^{15}N Chemical Shift values of K13W, K13T, and K13S GB3 variants

Residues in WT GB3		K13W GB3 ^{15}N (ppm)	K13W GB3 ^{1}H (ppm)	K13T GB3 ^{15}N (ppm)	K13T GB3 ^{1}H (ppm)	K13S GB3 ^{15}N (ppm)	K13S GB3 ^{1}H (ppm)
2	Gln	123.85	8.32	123.12	8.33	123.50	8.35
3	Tyr	124.30	9.03	124.29	9.04	124.33	9.04
4	Lys	122.60	9.09	122.76	9.07	122.67	9.08
5	Leu	126.71	8.59	126.81	8.61	126.83	8.63
6	Val	127.31	9.06	127.41	9.08	127.38	9.10
7	Ile	125.70	8.63	125.57	8.65	125.51	8.68
8	Asn	128.06	8.85	128.74	8.89	128.54	8.91
9	Gly	110.52	7.91	110.34	7.86	110.53	7.91
10	Lys	120.00	9.25	120.68	9.38	120.73	9.29
11	Thr	109.33	8.81	109.30	8.79	110.16	8.81
12	Leu	124.63	7.33	125.09	7.44	124.95	7.56
13	Lys	118.00	8.24	110.56	8.33	117.00	8.06
14	Gly	110.80	8.09	118.21	8.37	110.23	8.32
15	Glu	118.22	8.38	116.01	8.79	118.29	8.36
16	Thr	116.37	8.76	111.91	8.10	116.23	8.83
17	Thr	112.05	8.12	115.35	8.90	111.84	8.13
18	Thr	115.40	8.90	124.53	7.92	115.36	8.91
19	Lys	124.52	7.93	124.94	9.08	124.46	7.93
20	Ala	124.92	9.08	115.53	8.46	124.85	9.09
21	Val	115.56	8.46	115.50	7.31	115.66	8.48
22	Asp	115.59	7.32	121.31	8.31	115.58	7.32
23	Ala	121.33	8.31	119.30	8.40	121.32	8.31
24	Glu	119.27	8.40	117.79	8.34	119.27	8.41
25	Thr	117.81	8.35	125.48	7.24	117.65	8.36
26	Ala	125.49	7.24	116.86	8.02	125.50	7.26
27	Glu	117.45	8.34	117.46	8.33	117.55	8.35
28	Lys	116.55	7.17	116.55	7.17	116.60	7.18
29	Ala	122.40	7.21	122.41	7.22	122.42	7.22
30	Phe	119.95	8.58	120.00	8.58	119.96	8.59
31	Lys	123.04	9.02	123.11	9.03	123.04	9.05
32	Gln	119.85	7.49	119.86	7.50	119.92	7.52
33	Tyr	120.56	8.01	120.59	8.03	120.54	8.02
34	Ala	122.70	9.16	122.71	9.18	122.70	9.17
35	Asn	117.96	8.38	118.40	8.36	118.14	8.41
36	Asp	121.39	8.78	121.37	8.80	121.28	8.80
37	Gln	115.50	7.35	115.46	7.36	115.41	7.36

Residues in WT GB3		K13W GB3 ^{15}N (ppm)	K13W GB3 ^{1}H (ppm)	K13T GB3 ^{15}N (ppm)	K13T GB3 ^{1}H (ppm)	K13S GB3 ^{15}N (ppm)	K13S GB3 ^{1}H (ppm)
38	Gly	108.49	7.76	108.50	7.76	108.48	7.76
39	Val	120.96	8.09	120.99	8.12	120.98	8.13
40	Asp	128.19	8.69	128.37	8.73	128.27	8.70
41	Gly	107.47	7.92	107.44	7.89	107.46	7.93
42	Val	120.73	8.22	120.63	8.23	120.66	8.21
43	Trp	131.25	9.31	131.15	9.29	131.18	9.31
44	Thr	114.91	9.38	114.81	9.37	114.87	9.39
45	Tyr	120.52	8.55	120.45	8.54	120.48	8.56
46	Asp	128.51	7.54	128.56	7.54	128.51	7.53
47	Asp	125.12	8.57	125.13	8.56	125.14	8.58
48	Ala	120.06	8.32	120.04	8.32	120.08	8.33
49	Thr	135.68	6.99	135.66	6.99	135.69	7.00
50	Lys	123.23	7.85	123.22	7.84	123.24	7.85
51	Thr	111.39	7.37	111.44	7.38	111.45	7.38
52	Phe	131.47	10.36	131.41	10.37	131.44	10.37
53	Thr	117.95	9.13	117.78	9.12	117.97	9.14
54	Val	123.15	7.93	123.59	8.31	123.85	8.37
55	Thr	124.19	8.34	124.14	8.31	124.19	8.33
56	Glu	133.87	7.97	133.91	7.98	133.98	8.05

Supplementary Table 9. ^{1}H and ^{15}N Chemical Shift values of K13R, K13Q, and K13P GB3 variants

Residues in WT GB3		K13R GB3 ^{15}N (ppm)	K13R GB3 ^{1}H (ppm)	K13Q GB3 ^{15}N (ppm)	K13Q GB3 ^{1}H (ppm)	K13P GB3 ^{15}N (ppm)	K13P GB3 ^{1}H (ppm)
2	Gln	123.48	8.28	123.48	8.28	125.13	8.63
3	Tyr	124.33	9.04	124.33	9.04	124.35	9.02
4	Lys	122.72	9.08	122.72	9.08	122.09	9.07
5	Leu	126.76	8.62	126.76	8.62	126.79	8.74
6	Val	127.36	9.11	127.36	9.11	127.98	9.17
7	Ile	125.61	8.72	125.61	8.72	125.55	8.74
8	Asn	129.26	8.95	129.26	8.95	127.43	8.86
9	Gly	110.19	7.81	110.19	7.81	110.03	7.66
10	Lys	120.79	9.45	120.79	9.45	118.85	9.16
11	Thr	109.49	8.75	109.49	8.75	115.43	8.44
12	Leu	125.58	7.60	125.58	7.60	125.93	8.70
13	Lys	123.60	8.14	123.60	8.14	-	-
14	Gly	109.92	8.26	109.92	8.26	107.97	8.24
15	Glu	118.26	8.37	118.26	8.37	118.96	8.30
16	Thr	115.93	8.80	115.93	8.80	118.05	8.61
17	Thr	111.97	8.11	111.97	8.11	111.08	8.27
18	Thr	115.34	8.91	115.34	8.91	115.75	8.92
19	Lys	124.53	7.92	124.53	7.92	124.48	7.93
20	Ala	124.93	9.09	124.93	9.09	124.99	9.09
21	Val	115.55	8.46	115.55	8.46	117.26	8.48
22	Asp	115.56	7.31	115.56	7.31	114.55	7.24
23	Ala	121.32	8.31	121.32	8.31	121.31	8.32
24	Glu	119.29	8.41	119.29	8.41	119.33	8.41
25	Thr	117.62	8.34	117.62	8.34	117.69	8.41
26	Ala	125.49	7.24	125.49	7.24	125.59	7.32
27	Glu	117.62	8.34	117.62	8.34	117.57	8.38
28	Lys	116.58	7.17	116.58	7.17	116.55	7.25
29	Ala	122.41	7.21	122.41	7.21	122.46	7.28
30	Phe	119.98	8.58	119.98	8.58	120.07	8.59
31	Lys	123.10	9.02	123.10	9.02	122.40	9.03
32	Gln	119.89	7.50	119.89	7.50	120.36	7.66
33	Tyr	120.61	8.04	120.61	8.04	120.18	7.85
34	Ala	122.71	9.18	122.71	9.18	122.40	9.15
35	Asn	118.26	8.37	118.26	8.37	118.29	8.55
36	Asp	121.39	8.80	121.39	8.80	120.33	8.75
37	Gln	115.47	7.36	115.47	7.36	115.60	7.32

Residues in WT GB3		K13R GB3 ^{15}N (ppm)	^{1}H (ppm)	K13Q GB3 ^{15}N (ppm)	^{1}H (ppm)	K13P GB3 ^{15}N (ppm)	^{1}H (ppm)
38	Gly	108.48	7.77	108.48	7.77	108.26	7.60
39	Val	121.00	8.11	121.00	8.11	120.34	8.19
40	Asp	128.37	8.73	128.37	8.73	128.72	8.63
41	Gly	107.51	7.91	107.51	7.91	106.88	8.06
42	Val	120.54	8.21	120.54	8.21	120.98	8.22
43	Trp	131.22	9.31	131.22	9.31	131.31	9.35
44	Thr	114.76	9.37	114.76	9.37	115.14	9.42
45	Tyr	120.41	8.55	120.41	8.55	120.76	8.57
46	Asp	128.53	7.55	128.53	7.55	128.44	7.45
47	Asp	125.15	8.57	125.15	8.57	125.15	8.59
48	Ala	120.05	8.32	120.05	8.32	120.11	8.34
49	Thr	103.39	6.99	103.39	6.99	103.47	7.00
50	Lys	123.27	7.84	123.27	7.84	123.40	7.85
51	Thr	111.44	7.38	111.44	7.38	111.29	7.34
52	Phe	131.42	10.37	131.42	10.37	131.81	10.34
53	Thr	117.81	9.14	117.81	9.14	117.94	8.99
54	Val	123.48	8.28	123.48	8.28	123.12	8.32
55	Thr	124.35	8.34	124.35	8.34	124.45	8.46
56	Glu	133.92	7.88	133.92	7.88	134.17	8.24

Supplementary Table 10. ^{1}H and ^{15}N Chemical Shift values of K13L, K13G, and K13E GB3 variants

Residues in WT GB3		K13L GB3 ^{15}N (ppm)	K13L GB3 ^{1}H (ppm)	K13G GB3 ^{15}N (ppm)	K13G GB3 ^{1}H (ppm)	K13E GB3 ^{15}N (ppm)	K13E GB3 ^{1}H (ppm)
2	Gln	123.98	8.35	123.83	8.35	123.38	8.34
3	Tyr	124.29	9.04	124.05	9.04	124.27	9.03
4	Lys	122.60	9.07	122.19	9.06	122.58	9.06
5	Leu	126.66	8.57	126.46	8.62	126.75	8.60
6	Val	127.41	9.08	127.25	9.09	127.50	9.09
7	Ile	125.76	8.65	125.50	8.54	125.60	8.65
8	Asn	128.58	8.89	128.03	8.94	128.28	8.88
9	Gly	110.44	7.95	109.87	7.81	110.20	7.96
10	Lys	120.43	9.20	120.16	8.99	120.27	9.12
11	Thr	110.09	8.68	111.98	8.64	110.74	8.73
12	Leu	125.13	7.57	124.90	7.95	125.00	7.59
13	Lys	118.73	8.33	108.73	7.96	121.94	8.07
14	Gly	109.84	8.37	108.82	8.19	109.91	8.29
15	Glu	118.03	8.38	117.86	8.43	118.45	8.34
16	Thr	116.04	8.72	116.37	8.79	116.22	8.78
17	Thr	112.15	8.12	111.58	8.15	111.99	8.13
18	Thr	115.35	8.89	115.36	8.90	115.37	8.88
19	Lys	124.59	7.93	124.40	7.93	124.50	7.93
20	Ala	124.94	9.08	124.70	9.08	124.90	9.07
21	Val	115.53	8.46	115.33	8.45	115.58	8.46
22	Asp	115.64	7.32	115.31	7.30	115.66	7.33
23	Ala	121.32	8.32	121.02	8.31	121.33	8.32
24	Glu	119.29	8.41	119.07	8.39	119.28	8.41
25	Thr	117.83	8.34	117.25	8.34	117.86	8.35
26	Ala	125.48	7.24	125.22	7.27	125.50	7.26
27	Glu	117.44	8.34	117.68	8.35	117.42	8.35
28	Lys	116.55	7.17	116.33	7.19	116.57	7.18
29	Ala	122.41	7.22	122.15	7.23	122.42	7.22
30	Phe	119.92	8.58	119.76	8.57	119.98	8.58
31	Lys	123.11	9.03	122.58	9.05	123.01	9.04
32	Gln	119.86	7.50	119.91	7.55	119.94	7.52
33	Tyr	120.59	8.03	120.26	7.96	120.54	8.01
34	Ala	122.71	9.19	122.39	9.13	122.68	9.16
35	Asn	118.23	8.38	118.13	8.24	118.12	8.41
36	Asp	121.30	8.78	120.67	8.74	121.21	8.78
37	Gln	115.50	7.38	114.97	7.35	115.39	7.35

Residues in WT GB3		K13L GB3 ^{15}N (ppm)	^{1}H (ppm)	K13G GB3 ^{15}N (ppm)	^{1}H (ppm)	K13E GB3 ^{15}N (ppm)	^{1}H (ppm)
38	Gly	108.54	7.77	108.40	7.70	108.50	7.75
39	Val	120.99	8.13	120.88	8.16	121.00	8.13
40	Asp	128.18	8.70	128.04	8.65	128.36	8.71
41	Gly	107.49	7.93	107.08	7.93	107.45	7.92
42	Val	120.69	8.22	120.38	8.20	120.70	8.23
43	Trp	131.23	9.32	130.78	9.32	131.09	9.30
44	Thr	114.95	9.39	114.75	9.38	114.93	9.37
45	Tyr	120.54	8.56	120.35	8.55	120.56	8.55
46	Asp	128.50	7.53	128.29	7.50	128.50	7.52
47	Asp	125.13	8.57	124.89	8.56	125.12	8.57
48	Ala	120.04	8.32	119.82	8.32	120.06	8.33
49	Thr	135.66	6.99	135.64	7.01	135.70	6.99
50	Lys	123.21	7.85	123.22	7.84	123.20	7.85
51	Thr	111.44	7.38	111.24	7.36	111.45	7.37
52	Phe	131.41	10.35	131.22	10.35	131.39	10.34
53	Thr	118.04	9.14	117.91	9.13	117.99	9.13
54	Val	125.09	8.00	123.01	8.32	124.02	8.33
55	Thr	124.26	8.37	124.08	8.41	123.94	8.38
56	Glu	133.97	8.04	133.63	8.11	133.80	8.10

Supplementary Table 11. ^{1}H and ^{15}N Chemical Shift values of K13C, K13D, and K13I GB3 variants

Residues in WT GB3		K13C GB3 ^{15}N (ppm)	K13C GB3 ^{1}H (ppm)	K13D GB3 ^{15}N (ppm)	K13D GB3 ^{1}H (ppm)	K13I GB3 ^{15}N (ppm)	K13I GB3 ^{1}H (ppm)
2	Gln	123.07	8.28	124.00	8.34	124.04	8.29
3	Tyr	124.32	9.03	124.21	9.02	124.31	9.04
4	Lys	122.72	9.07	122.50	9.05	122.81	9.06
5	Leu	126.87	8.61	126.68	8.56	126.87	8.59
6	Val	127.37	9.08	127.48	9.07	127.53	9.09
7	Ile	125.54	8.68	125.68	8.57	125.79	8.66
8	Asn	128.69	8.90	127.84	8.85	128.41	8.86
9	Gly	110.97	7.84	110.53	8.13	110.61	7.92
10	Lys	120.40	9.29	120.18	9.14	119.88	9.28
11	Thr	110.04	8.78	110.81	8.73	109.29	8.71
12	Leu	124.81	7.51	124.64	7.57	125.69	7.50
13	Lys	121.10	8.18	122.26	8.06	123.97	7.97
14	Gly	110.93	8.37	108.23	8.32	112.45	8.47
15	Glu	118.37	8.37	118.52	8.30	118.67	8.37
16	Thr	116.07	8.80	116.18	8.72	115.89	8.78
17	Thr	111.90	8.11	112.19	8.12	112.05	8.10
18	Thr	115.34	8.90	115.37	8.86	115.28	8.89
19	Lys	124.59	7.92	124.63	7.94	124.60	7.93
20	Ala	125.03	9.07	125.06	9.05	125.04	9.07
21	Val	115.30	8.43	115.28	8.42	115.28	8.43
22	Asp	115.56	7.31	115.70	7.33	115.59	7.31
23	Ala	121.33	8.32	121.35	8.33	121.33	8.32
24	Glu	119.31	8.41	119.30	8.40	119.31	8.41
25	Thr	117.57	8.34	117.69	8.34	117.68	8.34
26	Ala	125.50	7.24	125.51	7.26	125.49	7.23
27	Glu	117.57	8.34	117.48	8.34	117.68	8.34
28	Lys	116.57	7.17	116.57	7.18	116.57	7.17
29	Ala	122.42	7.21	122.44	7.23	122.41	7.21
30	Phe	120.00	8.58	119.96	8.57	119.99	8.57
31	Lys	123.12	9.04	123.11	9.04	123.19	9.03
32	Gln	119.88	7.50	119.98	7.52	119.85	7.49
33	Tyr	120.58	8.02	120.55	8.01	120.41	7.96
34	Ala	122.71	9.16	122.75	9.17	122.69	9.17
35	Asn	118.15	8.38	118.12	8.38	118.16	8.36
36	Asp	121.34	8.80	121.17	8.75	121.37	8.81
37	Gln	115.42	7.35	115.42	7.38	115.47	7.36

Residues in WT GB3		K13C GB3 ^{15}N (ppm)	K13C GB3 ^{1}H (ppm)	K13D GB3 ^{15}N (ppm)	K13D GB3 ^{1}H (ppm)	K13I GB3 ^{15}N (ppm)	K13I GB3 ^{1}H (ppm)
38	Gly	108.36	7.75	108.55	7.75	108.54	7.76
39	Val	120.99	8.11	121.04	8.15	120.64	8.05
40	Asp	128.41	8.74	128.34	8.71	128.48	8.76
41	Gly	107.45	7.90	107.52	7.93	107.52	7.88
42	Val	120.64	8.22	120.72	8.22	120.61	8.22
43	Trp	131.13	9.29	131.04	9.29	131.07	9.29
44	Thr	114.82	9.37	115.00	9.37	114.76	9.36
45	Tyr	120.47	8.54	120.67	8.55	120.41	8.54
46	Asp	128.52	7.53	128.48	7.50	128.53	7.54
47	Asp	125.14	8.56	125.11	8.56	125.14	8.56
48	Ala	120.04	8.32	120.04	8.32	120.02	8.31
49	Thr	135.67	6.99	135.70	6.99	135.65	6.99
50	Lys	123.24	7.84	123.20	7.84	123.22	7.84
51	Thr	111.45	7.37	111.41	7.36	111.49	7.38
52	Phe	131.41	10.35	131.37	10.31	131.37	10.35
53	Thr	117.87	9.12	118.13	9.12	117.77	9.12
54	Val	123.77	8.36	124.00	8.34	121.05	8.12
55	Thr	124.09	8.30	124.14	8.39	123.67	8.35
56	Glu	133.90	8.05	133.74	8.15	133.82	8.00

Supplementary Table 12. ^{1}H and ^{15}N Chemical Shift values of K13V and K13M GB3 variants

Residues in WT GB3		K13V GB3 ^{15}N (ppm)	K13V GB3 ^{1}H (ppm)	K13M GB3 ^{15}N (ppm)	K13M GB3 ^{1}H (ppm)
2	Gln	124.06	8.28	123.36	8.30
3	Tyr	124.31	9.04	124.36	9.03
4	Lys	122.80	9.06	122.81	9.06
5	Leu	126.88	8.59	126.89	8.60
6	Val	127.52	9.09	127.46	9.09
7	Ile	125.78	8.67	125.71	8.68
8	Asn	128.33	8.87	128.82	8.90
9	Gly	110.66	7.93	110.57	7.84
10	Lys	120.01	9.31	119.90	9.20
11	Thr	109.34	8.72	109.90	8.72
12	Leu	125.60	7.47	125.23	7.57
13	Lys	122.89	7.96	122.68	8.09
14	Gly	112.12	8.45	110.05	8.35
15	Glu	118.62	8.38	118.40	8.35
16	Thr	115.84	8.79	116.06	8.79
17	Thr	112.00	8.09	111.94	8.11
18	Thr	115.29	8.89	115.31	8.89
19	Lys	124.59	7.92	124.45	7.92
20	Ala	125.04	9.08	124.90	9.07
21	Val	115.27	8.43	115.55	8.47
22	Asp	115.57	7.32	115.64	7.31
23	Ala	121.31	8.32	121.32	8.30
24	Glu	119.30	8.41	119.30	8.41
25	Thr	117.65	8.34	117.46	8.33
26	Ala	125.50	7.23	125.49	7.24
27	Glu	117.65	8.34	117.69	8.35
28	Lys	116.56	7.17	116.54	7.16
29	Ala	122.40	7.21	122.39	7.21
30	Phe	120.01	8.57	119.99	8.57
31	Lys	123.17	9.03	123.10	9.02
32	Gln	119.84	7.49	119.85	7.49
33	Tyr	120.63	8.05	120.54	8.02
34	Ala	122.68	9.17	122.70	9.16
35	Asn	118.14	8.36	118.15	8.38

Residues in WT GB3		K13V GB3 ^{15}N (ppm)	^{1}H (ppm)	K13M GB3 ^{15}N (ppm)	^{1}H (ppm)
36	Asp	121.37	8.81	121.28	8.79
37	Gln	115.45	7.36	115.44	7.35
38	Gly	108.52	7.76	108.49	7.75
39	Val	121.04	8.12	120.97	8.12
40	Asp	128.53	8.78	128.26	8.70
41	Gly	107.49	7.87	107.47	7.91
42	Val	120.60	8.22	120.65	8.21
43	Trp	131.06	9.29	131.17	9.30
44	Thr	114.75	9.37	114.82	9.37
45	Tyr	120.39	8.54	120.43	8.54
46	Asp	128.53	7.54	128.49	7.52
47	Asp	125.13	8.56	125.16	8.57
48	Ala	120.01	8.31	120.04	8.32
49	Thr	135.64	6.98	103.45	6.99
50	Lys	123.21	7.84	123.23	7.84
51	Thr	111.48	7.38	111.54	7.38
52	Phe	131.38	10.36	131.39	10.35
53	Thr	117.72	9.12	117.90	9.12
54	Val	123.61	8.34	123.75	8.34
55	Thr	124.00	8.29	124.19	8.32
56	Glu	133.78	7.99	133.96	8.02

References

1. Xu J. X., Alom M. S., Yadav R., Fitzkee N. C. Predicting protein function and orientation on a gold nanoparticle surface using a residue-based affinity scale. GitHub, 10.5281/zenodo.7272402, 2022.
2. Hubbard S., Thornton J. Naccess: Program for calculating accessibilities. *Department of Biochemistry and Molecular Biology, University College of London*, (1992).
3. Wang A., Vangala K., Vo T., Zhang D., Fitzkee N. C. A three-step model for protein–gold nanoparticle adsorption. *J. Phys. Chem. C* **118**, 8134-8142 (2014).
4. Woods K. E., Perera Y. R., Davidson M. B., Wilks C. A., Yadav D. K., Fitzkee N. C. Understanding protein structure deformation on the surface of gold nanoparticles of varying size. *J. Phys. Chem. C* **120**, 27944-27953 (2016).
5. Roach P., Farrar D., Perry C. C. Interpretation of protein adsorption: Surface-induced conformational changes. *J. Am. Chem. Soc.* **127**, 8168-8173 (2005).
6. Shao Q., Hall C. K. Binding preferences of amino acids for gold nanoparticles: A molecular simulation study. *Langmuir* **32**, 7888-7896 (2016).
7. Hoefling M., Iori F., Corni S., Gottschalk K.-E. Interaction of amino acids with the Au(111) surface: Adsorption free energies from molecular dynamics simulations. *Langmuir* **26**, 8347-8351 (2010).
8. Iori F., Di Felice R., Molinari E., Corni S. Golp: An atomistic force-field to describe the interaction of proteins with Au(111) surfaces in water. *J. Comput. Chem.* **30**, 1465-1476 (2009).
9. Power D., Rouse I., Poggio S., Brandt E., Lopez H., Lyubartsev A., et al. A multiscale model of protein adsorption on a nanoparticle surface. *Modell. Simul. Mater. Sci. Eng.* **27**, 084003 (2019).
10. Palafox-Hernandez J. P., Tang Z., Hughes Z. E., Li Y., Swihart M. T., Prasad P. N., et al. Comparative study of materials-binding peptide interactions with gold and silver surfaces and nanostructures: A thermodynamic basis for biological selectivity of inorganic materials. *Chem. Mater.* **26**, 4960-4969 (2014).
11. Walsh T. R. Pathways to structure–property relationships of peptide–materials interfaces: Challenges in predicting molecular structures. *Acc. Chem. Res.* **50**, 1617-1624 (2017).
12. Tavanti F., Pedone A., Menziani M. C. Competitive binding of proteins to gold nanoparticles disclosed by molecular dynamics simulations. *J. Phys. Chem. C* **119**, 22172-22180 (2015).
13. Petkova G. A., Záruba K., Žvátora P., Král V. Gold and silver nanoparticles for biomolecule immobilization and enzymatic catalysis. *Nanoscale Res. Lett.* **7**, 287 (2012).
14. Korkhin Y., Kalb A. J., Peretz M., Bogin O., Burstein Y., Frolow F. Nadp-dependent bacterial alcohol dehydrogenases: Crystal structure, cofactor-binding and cofactor specificity of the adhs of clostridium beijerinckii and thermoanaerobacter brockii11edited by r. Huber. *J. Mol. Biol.* **278**, 967-981 (1998).
15. Baruah P., Yesylevskyy S. O., Aguan K., Mitra S. Modulation of enzyme activity at nano-bio interface: A case study with acetylcholinesterase and citrate synthase adsorbed on colloidal metal nanoparticles. *J. Mol. Liq.* **325**, 115201 (2021).

16. Yu X., Sigler S. C., Hossain D., Wierdl M., Gwaltney S. R., Potter P. M., et al. Global and local molecular dynamics of a bacterial carboxylesterase provide insight into its catalytic mechanism. *J. Mol. Model.* **18**, 2869-2883 (2012).
17. Remington S. J. Structure and mechanism of citrate synthase. In: Stadtman ER, Chock PB (eds). *Current topics in cellular regulation*, vol. 33. Academic Press, 1992, pp 209-229.
18. Cans A. S., Dean S. L., Reyes F. E., Keating C. D. Synthesis and characterization of enzyme-Au bioconjugates: HRP and fluorescein-labeled HRP. *NanoBiotechnology* **3**, 12-22 (2007).
19. Mogharrab N., Ghourchian H., Amininasab M. Structural stabilization and functional improvement of horseradish peroxidase upon modification of accessible lysines: Experiments and simulation. *Biophys. J.* **92**, 1192-1203 (2007).
20. Macdonald I. D. G., Smith W. E. Orientation of cytochrome c adsorbed on a citrate-reduced silver colloid surface. *Langmuir* **12**, 706-713 (1996).
21. Tellechea E., Wilson K. J., Bravo E., Hamad-Schifferli K. Engineering the interface between glucose oxidase and nanoparticles. *Langmuir* **28**, 5190-5200 (2012).
22. Leskovac V., Trivić S., Wohlfahrt G., Kandrač J., Peričin D. Glucose oxidase from *Aspergillus niger*: The mechanism of action with molecular oxygen, quinones, and one-electron acceptors. *Int. J. Biochem. Cell Biol.* **37**, 731-750 (2005).
