# Supplementary material for: Predicting protein function and orientation on a gold nanoparticle surface using a residue-based affinity scale
Source: Nat Commun. 2022 Nov 27;13:7313. doi: 10.1038/s41467-022-34749-w (PMC9701677; doi:10.1038/s41467-022-34749-w)
Supplement: Supplementary file 2 — Reporting Summary [file 41467_2022_34749_MOESM2_ESM.pdf]

nature portfolio

Corresponding author(s): Nicholas C. Fitzkee

Last updated by author(s): Oct 25, 2022

Reporting Summary

Nature Portfolio wishes to improve the reproducibility of the work that we publish. This form provides structure for consistency and transparency in reporting. For further information on Nature Portfolio policies, see our Editorial Policies and the Editorial Policy Checklist.

Statistics

For all statistical analyses, confirm that the following items are present in the figure legend, table legend, main text, or Methods section.

n/a	Confirmed	
☐	☒	The exact sample size (*n*) for each experimental group/condition, given as a discrete number and unit of measurement
☐	☒	A statement on whether measurements were taken from distinct samples or whether the same sample was measured repeatedly
☒	☐	The statistical test(s) used AND whether they are one- or two-sided<br>*Only common tests should be described solely by name; describe more complex techniques in the Methods section.*
☒	☐	A description of all covariates tested
☒	☐	A description of any assumptions or corrections, such as tests of normality and adjustment for multiple comparisons
☐	☒	A full description of the statistical parameters including central tendency (e.g. means) or other basic estimates (e.g. regression coefficient) AND variation (e.g. standard deviation) or associated estimates of uncertainty (e.g. confidence intervals)
☒	☐	For null hypothesis testing, the test statistic (e.g. *F*, *t*, *r*) with confidence intervals, effect sizes, degrees of freedom and *P* value noted<br>*Give P values as exact values whenever suitable.*
☒	☐	For Bayesian analysis, information on the choice of priors and Markov chain Monte Carlo settings
☒	☐	For hierarchical and complex designs, identification of the appropriate level for tests and full reporting of outcomes
☐	☒	Estimates of effect sizes (e.g. Cohen's *d*, Pearson's *r*), indicating how they were calculated

Our web collection on statistics for biologists contains articles on many of the points above.

Software and code

Policy information about availability of computer code

Data collection	NMR data were collected using TopSpin 3.6.4, which is freely available from Bruker Biospin.
Data analysis	Images were generated with PyMOL version 2.5.0. Statistical analysis was performed using OriginPro 2021. GeneRunner and the New England BioLabs site (https://tmcalculator.neb.com/#!/main) were used to calculate melting temperatures for primers, and surface area calculations were performed using NACCESS. NMR data were processed using NMRPipe and TopSpin. NMR data were visualized using NMRFAM Sparky. The alpha value surface prediction is implemented in Python 3.8 and freely available at our GitHub site and linked in the text (https://github.com/FitzkeeLab/citrate-aunp-predict.)

For manuscripts utilizing custom algorithms or software that are central to the research but not yet described in published literature, software must be made available to editors and reviewers. We strongly encourage code deposition in a community repository (e.g. GitHub). See the Nature Portfolio guidelines for submitting code & software for further information.

Data

Policy information about availability of data

All manuscripts must include a data availability statement. This statement should provide the following information, where applicable:

- Accession codes, unique identifiers, or web links for publicly available datasets
- A description of any restrictions on data availability
- For clinical datasets or third party data, please ensure that the statement adheres to our policy

Protein structures used in this study were obtained from the Protein Data Bank (https://www.rcsb.org/). PDB accession codes are: 2OED (GB3), 5B1E (proteinase K), 1CA2 (carbonic hydrase), 1HCH (horseradish peroxidase), 4ewz (insulin), 1YKF (alcohol dehydrogenase), 1EEA (acetylcholinesterase), 1CTS (citrate synthase), 1OCD (cytochrome c), 1GAL (gluocose oxidase), and 3GHG (fibrinogen). All data, including NMR kinetics data, UV-vis spectra, protein activity data, and surface interaction calculations data generated in this study have been deposited in the Zenodo database under accession code https://doi.org/10.5281/zenodo.7272338.

Human research participants

Policy information about studies involving human research participants and Sex and Gender in Research.

Reporting on sex and gender	not applicable
Population characteristics	not applicable
Recruitment	not applicable
Ethics oversight	not applicable

Note that full information on the approval of the study protocol must also be provided in the manuscript.

Field-specific reporting

Please select the one below that is the best fit for your research. If you are not sure, read the appropriate sections before making your selection.

☒ Life sciences ☐ Behavioural & social sciences ☐ Ecological, evolutionary & environmental sciences

For a reference copy of the document with all sections, see nature.com/documents/nr-reporting-summary-flat.pdf

Life sciences study design

All studies must disclose on these points even when the disclosure is negative.

Sample size	All samples were performed with N = 3 unless otherwise specified in the text. N = 3 was chosen because it is sufficient to generate a standard deviation and confidence interval and provided a sufficient means for comparing the observations.
Data exclusions	No data were excluded.
Replication	All samples were created independently, and the average and 95% confidence interval of three trials are reported. All attempts at replication were successful and included in the results.
Randomization	This is not a drug treatment study; randomization is not applicable.
Blinding	This is not a drug treatment study; blinding is not applicable.

Reporting for specific materials, systems and methods

We require information from authors about some types of materials, experimental systems and methods used in many studies. Here, indicate whether each material, system or method listed is relevant to your study. If you are not sure if a list item applies to your research, read the appropriate section before selecting a response.

Materials & experimental systems

n/a	Involved in the study
☒	☐ Antibodies
☒	☐ Eukaryotic cell lines
☒	☐ Palaeontology and archaeology
☒	☐ Animals and other organisms
☒	☐ Clinical data
☒	☐ Dual use research of concern

Methods

n/a	Involved in the study
☒	☐ ChIP-seq
☒	☐ Flow cytometry
☒	☐ MRI-based neuroimaging
